# A Dual Receptor Crosstalk Model of G-Protein-Coupled Signal Transduction

**DOI:** 10.1371/journal.pcbi.1000185

**Published:** 2008-09-26

**Authors:** Patrick Flaherty, Mala L. Radhakrishnan, Tuan Dinh, Robert A. Rebres, Tamara I. Roach, Michael I. Jordan, Adam P. Arkin

**Affiliations:** 1 Department of Electrical Engineering and Computer Sciences, University of California Berkeley, Berkeley, California, United States of America; 2 Computer Science and Artificial Intelligence Laboratory, Department of Chemistry, Massachusetts Institute of Technology, Cambridge, Massachusetts, United States of America; 3 Department of Bioengineering, University of California Berkeley, Berkeley, California, United States of America; 4 Alliance for Cellular Signaling, Northern California Institute for Research and Education and the University of California, Veterans Affairs Medical Center, San Francisco, California, United States of America; 5 Department of Statistics and Computer Science Division, University of California Berkeley, Berkeley, California, United States of America; 6 Physical Biosciences Division, Lawrence Berkeley Laboratory, Berkeley, California, United States of America; 7 Howard Hughes Medical Institute, University of California Berkeley, Berkeley, California, United States of America; 8 Department of Bioengineering, University of California Berkeley, Berkeley, California, United States of America; California Institute of Technology, United States of America

## Abstract

Macrophage cells that are stimulated by two different ligands that bind to G-protein-coupled receptors (GPCRs) usually respond as if the stimulus effects are additive, but for a minority of ligand combinations the response is synergistic. The G-protein-coupled receptor system integrates signaling cues from the environment to actuate cell morphology, gene expression, ion homeostasis, and other physiological states. We analyze the effects of the two signaling molecules complement factors 5a (C5a) and uridine diphosphate (UDP) on the intracellular second messenger calcium to elucidate the principles that govern the processing of multiple signals by GPCRs. We have developed a formal hypothesis, in the form of a kinetic model, for the mechanism of action of this GPCR signal transduction system using data obtained from RAW264.7 macrophage cells. Bayesian statistical methods are employed to represent uncertainty in both data and model parameters and formally tie the model to experimental data. When the model is also used as a tool in the design of experiments, it predicts a synergistic region in the calcium peak height dose response that results when cells are simultaneously stimulated by C5a and UDP. An analysis of the model reveals a potential mechanism for crosstalk between the Gαi-coupled C5a receptor and the Gαq-coupled UDP receptor signaling systems that results in synergistic calcium release.

## Author Summary

The G protein signal transduction system transmits a wide variety of extracellular signals including light, odors, and hormones, to intracellular effectors in diverse cell types in eukaryotes. G-protein-coupled receptors are involved in many diseases including inflammation, cardiac dysfunction, and diabetes, and are the targets of 40–50% of modern drugs. Despite the physiological and pharmacological importance of this signal transduction system it is not known how the system buffers and integrates information at a biochemical level. The multiple receptors expressed by every cell pass their signals through a common set of downstream effectors distinguished by multiple isoforms with slightly different specificities and activities. The coupling among these pathways causes interactions among the signals sent by the different classes of receptors. We have developed a mechanistic model of the G protein signal transduction system from the receptor to the central intracellular second-messenger calcium. We have used statistical methods to integrate a diverse set of experimental data into our model and quantify confidence in our model predictions. We used this model, trained on single receptor data, to predict the signal processing of two G-protein-coupled-receptor signals. Validation experiments support our hypothesized mechanism for dual receptor signal processing and the predictions of the model.

## Introduction

The G-protein-coupled signal transduction system integrates a wide range of intercellular signals and actuates downstream pathways. G-protein-coupled receptors (GPCRs) are composed of seven α-helices that span the plasma membrane, an extracellular domain that is activated by an agonist and an intracellular domain that binds a guanine nucleotide heterotrimer made up of different α, β, and γ subunit isoforms. This receptor system accounts for 40–50% of modern medicinal drug targets but only 10% of the known receptors are targeted by drugs [1]. Though the system is physiologically and pharmacologically important, the mechanism by which the system integrates multiple signals is not well understood [2].

We address the G-protein-mediated route to calcium release in RAW264.7 cells. When activated by a specific ligand, the G protein heterotrimer dissociates to free Gα-GTP and Gβγ. Specific Gα and Gβγ isoforms are able to bind specific isoforms of phospholipase C β (PLCβ) and catalyze the synthesis of inositol (1,4,5)-triphosphate (IP3) and diacylglycerol (DAG) from phosphatidylinositol (4,5)-bisphosphate (PIP2) [3],[4]. In addition to its catalytic activity, PLCβ acts as a GTPase for Gα-GTP [5]. IP3 binds to specific receptor-channels on the membrane of the ER to release Ca^2+^ into the cytosol [6]. DAG and Ca^2+^ bind to and activate protein kinase C (PKC) which may phosphorylate and inactivate specific PLCβ isoforms [7]. G protein receptor kinase (GRK) is activated once it is phosphorylated by PKC [8] and is localized to the plasma membrane by Gβγ [9]. Though phosphorylation has not been shown to be necessary for GRK activation, we have assumed so in our model because phosphorylation by PKC may release the inhibition of GRK2 by being bound to calmodulin [8]. Activated GRK can then phosphorylate specific GPCRs which leads to receptor inactivation—perhaps directly or by arrestin activity [8]. In this complex signal transduction network, Gα and Gβγ subunits have different patterns of specificity for PLCβ isoforms and calcium is an important cofactor in several important feedback loops [10].

The two extracellular signaling ligands we consider here are C5a and UDP. The small peptide C5a is a potent anaphylotoxin and a strong chemoattractant for many immune system components [11]. The calcium response due to stimulation by C5a is predominantly coupled through Gαi-linked heterotrimers. Macrophage cells and their precursors, monocytes, express several receptors that are specific to extracellular nucleotides and it has been shown that the P2Y6 receptor, which is sensitive to UDP, regulates the production and secretion of the chemokine interleukin 8 (IL-8) in monocytes [12]. The UDP response is mediated by Gαq-linked heterotrimers, but other receptors in the P2Y family may respond to UDP and couple the signal through other G protein isoforms [13].

Four recent models have sought to explore various aspects of the G protein coupled signal transduction system in detail. Lukas et al. compare measured calcium response over a range of bradykinin doses to their model predictions [14]. Mishra and Bhalla built a model to investigate the role of IP4 as a signal coincidence detector in the GPCR pathway [15]. The model by Lemon et al. predicts the calcium response to UTP stimulation and is the closest in focus to our model [16]. A recent model of calcium dynamics in RAW cells has been proposed that is quite similar to this model, but does not deal with crosstalk between receptors or formal statistical uncertainty in model predictions [17],[18].

Several hypotheses for the mechanism of crosstalk and synergy among GPCR-mediated pathways have been proposed. Crosstalk among GPCR-mediated pathways is important both physiologically and pharmaceutically. Quitterer et al. propose that crosstalk is mediated by Gβγ exchange between Gαi-coupled and Gαq-coupled receptors [19]. Zhu et al. speculated that PLC is under either conditional or dual regulation of Gβγ and Gα [20]. Though these hypothetical mechanisms for crosstalk among G protein coupled receptor systems are conceptually plausible we have not found these or any other of the many competing hypothetical mechanisms tested in the context of a quantitative mathematical model [2].

In this paper Bayesian statistical inference is used to provide a rigorous connection between the mathematical model derived from mass-action kinetics, prior information from in-vitro biochemical studies and heterogeneous experimental data. The prior distribution over the parameters represents our uncertainty before observing a set of experimental data. A broad, high variance, prior distribution means we are quite uncertain and a concentrated, low variance, prior means we are more certain about the parameter a priori. The objective of our inference is the posterior distribution over the parameters because it is an informed estimate of both the value of the parameter and the uncertainty in the parameter value. The posterior distribution over the parameters is then used as a tool for experiment design to estimate the model-based posterior distribution over observable quantities such as the cytosolic calcium concentration and to drive the design of new experiments. This statistical approach is possible in a model of this size because of the abundance and quality of the data collected for this study.

## Results

**Figure 1:**
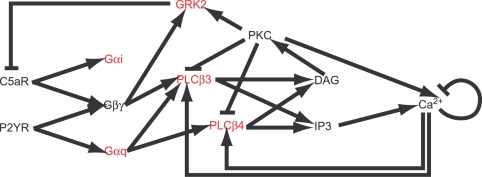
The model for crosstalk between the Gαi and Gαq pathways depends on both differential specificity and activity for Gαi, Gαq, and Gβγ interactions with PLCβ3 and PLCβ4 to catalyze PIP2 hydrolysis and calcium-dependent feedback control mediated by GRK and PKC. Selected model parameters are informed by calcium measurements taken for various ligand doses on wild-type and cell lines with shRNAi knockdowns on the proteins shown in red.

There are two main features of the structure of our model, shown in Figure 1, which contribute to crosstalk in the system and produce the key dynamical features in the calcium response: isoform specificity and calcium-dependent feedback. As we will show, by including multiple isoforms of PLCβ and Gα as well as the negative feedback mediated by PKC, GRK and the IP3 receptor itself, we are able to predict the synergistic interaction between C5a and UDP observed in the experimental data.

Our representation of the G-protein-coupled signal transduction system includes C5a and P2Y6 receptors, Gαi2, Gαq, Gβγ, PLCβ3, PLCβ4, PIP2, DAG, IP3, PKC, GRK2, calcium buffer, a Na^2+^/Ca^2+^ exchanger, a sarco(endo)plasmic reticulum Ca^2+^-ATPase (SERCA) pump, IP3 receptors and RGS. The model is composed of 53 coupled ordinary differential equations with 84 parameters and 24 non-zero initial conditions. The complete model equations are shown in Figure S7 and a more detailed model diagram is shown in Figure S6. The parameters and initial conditions are in Table S2 and Table S1, respectively. Where available, we have relied on *in-vitro* or *in-vivo* biochemical experiments for the reactions and parameter values (see Supporting Information). In cases where the biochemical parameter values were not known, we chose physically reasonable values. Twenty of the 84 parameters most relevant to the knock-down and wild-type data were estimated from cytosolic calcium measurements as described in the Methods section. Most reactions were assumed to be governed by mass-action kinetics, but for a few proteins—such as RGS—the mechanism of regulation is not known in enough detail and we have approximated with Michaelis-Menten kinetics or a phenomenological function.

We briefly discuss the reactions involving the Na^2+^/Ca^2+^ exchanger, SERCA pump, IP3 receptors, RGS and calcium buffer because they are important for the faithful representation of the system in our model. Regulators of G protein signaling (RGS) are GTPase proteins that down-regulate the extent of signaling [21]; RGS2 at least is expressed in RAW264.7 macrophage cells and therefore an RGS activity is included in our model. The mechanism of activation of RGS2 as it relates to Gαi and Gαq signaling is not entirely known and is difficult to assess because antibodies that specifically recognize RGS2 are not widely available [22]–[24]. We have assumed constitutive activity and expect as more information becomes available a more accurate model of the regulation of RGS2 and other RGS isoforms will be possible. The SERCA pump helps to bring the cytosolic Ca^2+^ concentration back to the resting level after stimulation. We have modeled the SERCA pump as in the Keizer and DeYoung model [25]. The IP3 dependent opening of ER calcium channels was found to be cooperative [26] and we have used the Meyer and Stryer model for the IP3-gated channel with a Hill coefficient of four [25],[27]. Finally, many other proteins such as calmodulin and the fluorescent indicator Fura-2 bind Ca^2+^. Because our measurements reflect these effects, we have included a general buffer for cytosolic calcium.

### Isoform Specificity

Complement factor 5a activates the C5a receptor which is a Gαi-coupled receptor [28]. The released Gβγ dimer activates PLCβ2 and PLCβ3 which are lumped and called PLCβ3 in our model because: (i) the activity of Gβγ-activated PLCβ3 has been shown to be greater than Gβγ-activated PLCβ2 in *in-vitro* studies and (ii) Gαq activates both PLCβ2 and PLCβ3 so the structural connections from Gβγ and Gαq to PLCβ2 and PLCβ3 in the model are identical [4],[29]. PLCβ1 is activated by Gβγ and Gαq, but RAW264.7 macrophage cells do not express this isoform, so we have not included it in the model. PLCβ3 then catalyzes the hydrolysis of phosphatidylinositol (4,5)-bisphosphate (PIP2) into inositol 1,4,5-trisphosphate (IP3) and diacylglycerol (DAG).

UDP stimulates the P2Y6 receptor and the associated Gαq-GTP activates both PLCβ3 [30] and PLCβ4 [31]. The GTPase rate of Gαq is increased 1000-fold when bound to PLCβ [5]. Due to this rapid hydrolysis rate, we have assumed, in our model, that PLCβ3 or PLCβ4 bound Gαq-GTP may only hydrolyze one molecule of PIP2 before releasing Gαq-GDP. Additionally, the Gβγ released by the P2Y6 receptor also activates PLCβ3 [30], but does not activate PLCβ4 [32].

Our model assumes that PLCβ3 does not simultaneously bind Gβγ and Gαq. Indeed, a biochemical study of PLCβ2 activity in reconstituted membrane fractions strongly argues that Gαq and Gβγ do not simultaneously bind this effector [33]. While this was specifically demonstrated for PLCβ2, we implicitly assume the same holds for PLCβ3 because we lump the two in our model. This is a mechanistic assumption of our model and an interesting issue for future testing with directed experiments.

### Calcium-Dependent Feedback

Though important for response specificity, the dynamical control of calcium release is not limited to the forward pathway in this system. Calcium participates in feedback processes that both enhance and inhibit its own release at multiple points in the pathway. There are four main nodes of calcium-dependent feedback control in our model: PLCβ, IP3 receptor, protein kinase C (PKC) and G protein receptor kinase (GRK).

Calcium enhances its own release by binding to the EF-hand domain on PLCβ and is required for PLCβ to hydrolyze PIP2 into IP3 and DAG [34]. Because the dissociation constant for PLCβ-Ca^2+^ in our model is larger than the basal concentration of cytosolic calcium, as more Ca^2+^ is released from the ER, more PLCβ-Ca^2+^ becomes available to bind Gαq or Gβγ. This positive feedback mechanism accelerates the release of Ca^2+^.

In our model, Ca^2+^ and IP3 cooperatively open the channel between the ER and the cytosol. It is believed that Ca^2+^ initially stimulates the IP3 receptor with maximal stimulatory effect at 100–300 nM [6]. At higher concentrations, Ca^2+^ has an inhibitory effect. We use the IP3 receptor model structure in the Keizer and DeYoung model for this component [25].

Protein kinase C (PKC) has been shown to phosphorylate PLCβ3 which inhibits PLCβ3 activation due to Gαq and Gβγ [35],[36]. PKC is activated when bound to DAG and Ca^2+^ [7],[37]. Because the preferred order of binding is not entirely known, PKC, DAG and Ca^2+^ form a thermodynamic cycle of reversible reaction with only the PKC-DAG-Ca^2+^ form active. In our model, the dissociation constant of PKC and Ca^2+^ is much greater than the basal Ca^2+^ concentration, and upon binding DAG, the PKC-DAG complex has a higher affinity for Ca^2+^ making the order of binding preferentially PKC to DAG then PKC-DAG to Ca^2+^. It is not known whether PLCβ4 is also regulated by PKC. We have assumed, in our model, the same mechanism of PKC regulation of PLCβ3 and PLCβ4.

The final key calcium-dependent feedback loop in our model is mediated by G protein receptor kinase (GRK). GRK2 phosphorylates and inactivates ligand-bound C5a receptors when activated by PKC and Gβγ. In sequence, PKC phosphorylates GRK2 which causes translocation to the plasma membrane [8]. When properly localized, GRK2 may bind Gβγ and then phosphorylate the C5a-C5a receptor complex to inactivate it [38]. This simplified representation of the receptor desensitization mechanism does not include arrestin activity, multiple receptor phosphorylation sites and other fine grain or slower biochemical interactions that may be present *in-vivo*.

### Single Ligand Experiments

Having specified the structure of our model, we direct our attention to the parameters. We estimate 20 of the 84 parameters in our model using a dataset composed of 96 Fura-2 time series measurements as described in the Materials and Methods section. Each experiment consists of 3–4 samples from different wells in a 96 well plate. There are 15 experiments spanning 9 doses of C5a and 14 experiments spanning 11 doses of UDP on wild-type cells in the dataset (see Figure S3). The dataset also contains calcium measurements on 5 different shRNAi knockdown cell lines constructed by lentiviral infection (see Figure S4). The time interval between samples is approximately 3–4 seconds and each time series is approximately 100–300 seconds of post-stimulation data. Table 1 shows a summary of the knockdown data used for statistical parameter estimation for this model in addition to the wild-type experiments.

**Table 1:** Dataset used for parameter estimation.

Cell Line	Measured Fraction Knockdown		Model Value			Sample Size					
						C5a			UDP		
	qRT-PCR	Western	Nominal	Lower	Upper	<10 nM	10–100 nM	>100 nM	<1 µM	1–10 µM	>10 µM
Wild-type	–	–	–	–	–	4	8	3	5	5	4
GRK2 (2)	90%±7%, *n* = 5	40%±6%, *n* = 6	40.0%	22.0%	58.0%	2	12	2	3	1	5
Gai2 (3)	83%±5%, *n* = 4	73%±6%, *n* = 5	73.0%	55.0%	91.0%	–	5	–	5	–	7
Gaq (3)	70%±8%, *n* = 7	66%±23%, *n* = 2	66.0%	0.0%	95.0%	–	3	–	1	–	3
PLCβ3 (1)	–	83%±15%, *n* = 3	83.0%	38.0%	100.0%	–	3	–	–	–	3
PLCβ4 (1)	87%±6%, *n* = 5	–	87.0%	69.0%	100.0%	–	4	–	4	–	4

Five different cell lines that have a perturbation in the level of a key signal transduction protein were constructed by shRNAi lentiviral infection. The calcium response from these cell lines in addition to the wild-type cell line were used to fit relevant parameters in the model. Because shRNAi does not entirely remove the protein product, the fraction knockdown was estimated by qRT-PCR and by Western blot analysis. The standard error (se) was computed for each estimate and the upper and lower confidence intervals were computed as ±3·se. The knockdown confidence intervals are used in the GPCR model to construct prediction confidence intervals for the calcium response. Where several cell lines were constructed for each knockdown, the best was selected and reported in parenthesis. The sample size for each knockdown-ligand dose combination is shown in the last 6 columns.

We find that our model is generally quantitatively consistent with the experimental data within measurement uncertainty. Where the model is less consistent with the data – specifically for the GRK knockdown experiment – we find the deviation has a reasonable biological explanation. The summary of the dataset and the fit of the model to each single ligand experiment are available in the Supporting Information. We briefly discuss some issues relating to goodness of fit and the Bayesian parameter estimation here.

**Figure 2:**
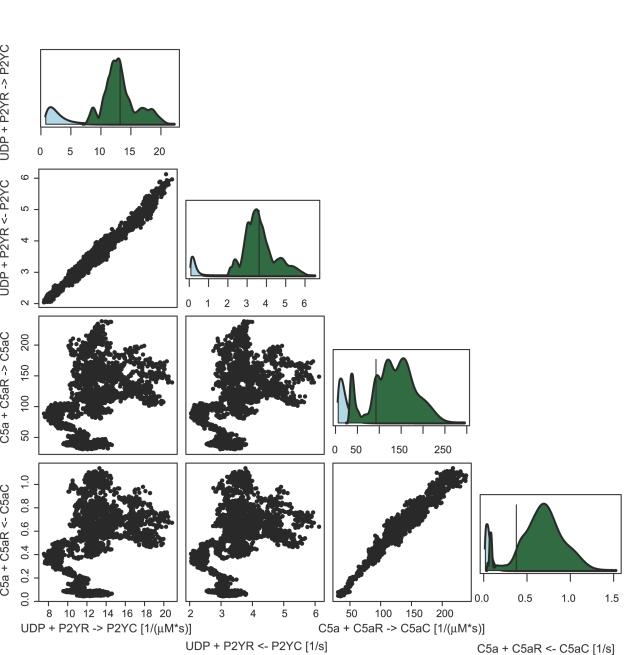
This figure shows that the single and pairwise marginal posterior distributions for the ligand binding reactions for the P2YR and C5aR receptors. The vertical line in the single marginal posterior distributions shows the point estimate that were selected. The posterior distributions show the dissociation constants for the reactions are tightly constrained by the data, while the values of the forward and reverse rates that make up the ratio are not as well constrained by the data. Additionally, as expected the UDP binding rates are not correlated with the C5a binding rates. Marginal posterior distributions for all parameters and a discussion of the point estimate selection can be found in Figure S2.

While most optimization procedures produce a point estimate of the parameters that maximize the goodness of fit of the model to the observed data, the Bayesian procedure we have employed here estimates the entire posterior distribution of the parameters given the data. This information is valuable for qualitatively and quantitatively evaluating the precision of the parameters estimates. Figure 2 shows, as a qualitative evaluation, that while the a-priori forward and reverse binding rates for the receptors (C5aR and P2YR) are uncorrelated they are correlated in the posterior distribution. The calcium measurements have informed and constrained the posterior estimates of the dissociation constants to be approximately 5 nM and 250 nM for the C5aR and P2YR respectively. We have quantitatively computed marginal highest posterior density (HPD) confidence intervals for each of the twenty parameters we have estimated from the data. Those estimates are shown in Table S3. Those parameters with large HPD intervals are not well informed by the measurements and are candidates for directed biochemical experiments.

### Wild-Type Experiments

**Figure 3:**
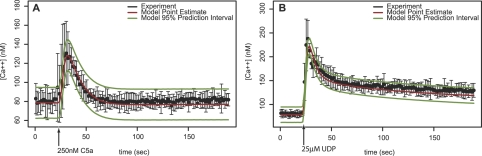
Model simulations are compared to experimental data. The point estimate is computed using the posterior distribution of the parameter as estimated by Markov chain Monte Carlo given the data from 96 experiments on C5a and UDP at various doses in combination with 5 different shRNAi knockdown cell lines. The 95% posterior predictive intervals are estimated by Monte Carlo simulations including both parameter and measurement uncertainty. The measured mean and approximate 95% confidence intervals of four replicates is shown by a black dot and error bar. (A) C5a at 250 nM was introduced at 20s and the experimentally observed pulse in cytosolic calcium concentration is shown. (B) The qualitative shape of the calcium pulse for 25 µM UDP is different than for 250 nM C5a. The pulse does not completely adapt and return to the prestimulated level. For both ligands, the model prediction confidence intervals overlap the data error bars that indicate the model fit is consistent with the data within the measurement uncertainty.

The calcium response to C5a adapts and returns to the basal level, but the UDP response has a sustained elevated calcium level that slowly decays. Figure 3 shows two representative experiments of the response of the wild-type cell to stimulation with C5a and UDP. We expect that the fit to this data will be good because 20 key model parameters were fit using an experimental dataset that included these experiments – the fit is indeed accurate. The point estimate curve is constructed from the maximum a-posteriori parameters from an MCMC chain. The prediction intervals are estimated by Monte Carlo sampling from the posterior parameter distribution and the measurement error distribution conditional on the parameters. The prediction confidence intervals generally cover the observed data.

### Knockdown Experiments

Lentiviral infection is used to introduce small hairpin RNAs to interfere with the translation of the key signaling proteins GRK2, Gαi2, Gαq, PLCβ3 and PLCβ4 [39]. There are three main sources of uncertainty in the knockdown experiment model predictions: parametric uncertainty, measurement uncertainty and knockdown efficiency uncertainty. We have dealt with the first two sources in the previous section on wild-type experiments. Here we address prediction variability due to knockdown efficiency uncertainty by using nominal parameter values.

**Figure 4:**
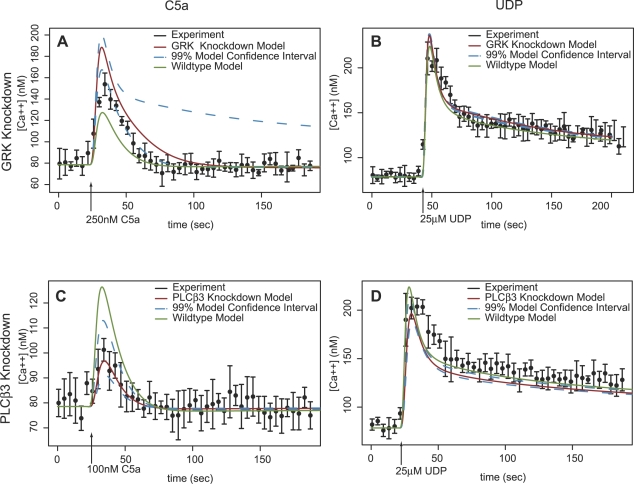
The model simulation results for GRK and PLCβ3 knockdown cell lines stimulated with C5a and UDP are shown. The experimental mean±1 s.d. of 3–4 replicates within one experimental run is shown in black. The knockdown simulation result with nominal knockdown fraction and parameters is shown in red and the wild-type simulation result is shown in green for comparison. Upper and lower model 99% confidence intervals (shown as blue dashed lines) are simulated using the upper and lower knockdown fraction values from Table 1. As expected the Ca^2+^ response to C5a in the GRK knockdown line (A) was increased compared to wild-type. The quantitative deviation between the model and data is possibly due to the availability of multiple redundant GRK isoforms. (B) The expected effect of the GRK knockdown on the UDP response is an increase in the cytosolic calcium levels. Because GRK2 does not directly desensitize the P2Y receptor in this model, the effect is likely due to a reduction of sequestration of Gβγ by GRK. (C) The signal transduction of the C5a response is predominantly through the PLCβ3 isoform. The effect of the PLCβ3 knockdown is much greater for C5a than for UDP (D).

Figure 4 shows simulations and experimental data for three representative knockdown experiments. The upper-left panel of Figure 4 shows a GRK knockdown line stimulated with 250 nM C5a. Because GRK2 desensitizes the C5a receptor, we expect that by eliminating the feedback mechanism, the calcium peak will be higher and more sustained. The experimental data as well as the model indeed show that effect. Quantitatively, the model prediction shows a greater effect than the experimental data. A likely reason is that the model only considers one isoform of GRK while there are four isoforms expressed in the RAW264.7 cell line (GRK1,2,4,6). If more than one isoform can desensitize the C5a receptor, the effective knockdown in desensitization function will be less than as measured by western blot analysis on GRK2.

While GRK does not desensitize the P2Y receptor in our model, it is a buffer for Gβγ released from Gαq. Reducing the amount of GRK will shift the equilibrium towards more Gβγ bound to PLCβ3 and thus more calcium release even though GRK does not directly feed back on the P2Y6 receptor. The top-right panel in Figure 4 shows that, based on the model, the peak intracellular calcium concentration is expected to be very slightly higher in the GRK2 knockdown line when stimulated by 25 µM UDP. A comparison of the experimental peak heights of the wild-type and GRK knockdown cell line data by t-test cannot reject the null hypothesis that the peak heights are equal (*p* = 0.9963). The effect of the GRK knockdown is expected to be so slight that the effect size is overwhelmed by the measurement error in the data. The effect of the uncertainty in the GRK2 knockdown fraction impacts the range of the confidence intervals of the predicted C5a response much more than the confidence intervals of the predicted UDP response which is consistent with GRK2 being a more significant component of the C5a response.

Our model structure has PLCβ3 stimulated by either Gβγ or Gαq. Because the C5a response signals only through PLCβ3 the effect of the knockdown is expected to be more pronounced for the C5a response than for the UDP response. The bottom-left panel of Figure 4 confirms that the model prediction is consistent with the representative experiment. The UDP response activates PLCβ3 through Gβγ, but also activates PLCβ3 and PLCβ4 with Gαq. Therefore, we expect that the calcium response should be more robust to perturbations in just one of the PLCβ isoforms. The UDP response in the PLCβ3 knockdown line (bottom right panel of Figure 4) shows that our model predicts the knockdown effect to be small relative to the total magnitude of the response in part due to the redundancy in the use of PLCβ isoforms in the UDP response.

Because this dataset was used for parameter estimation, the fit of model to the data may overstate the accuracy of the model. Nonetheless, the good fit does suggest that the model warrants being tested in truly predictive experiments; we describe such experiments in the following section.

### Double Ligand Experiments

We examine our model response to a simultaneous stimulation by C5a and UDP because it has been shown experimentally that macrophage cells respond synergistically to such conditions [40]. To quantify the amount of synergy or non-additivity that is present in the calcium response, a *synergy ratio* is computed for each ligand dose pair. The numerator of the ratio is the peak offset from baseline of the intracellular calcium concentration. The denominator of the ratio is the sum of the peak offsets when the cell or model is stimulated with only one ligand. A synergy is present when the ratio is greater than one implying the peak height is greater than expected from an additive combination of ligand effects. While this is certainly not the only possible measure of synergy it is widely adopted and has been used in previous studies on calcium synergy [40].

**Figure 5:**
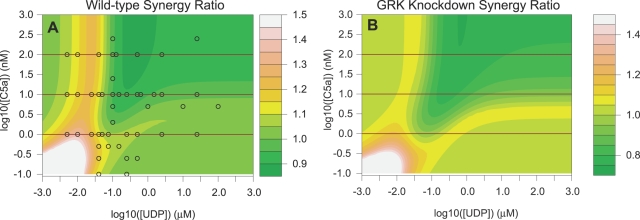
The model is used as a predictive tool to infer the effect of stimulating the cell simultaneously with UDP and C5a that signal through the Gαq and Gαi pathways, respectively. Synergy was measured as the ratio of peak height offset from baseline attained from simultaneous stimulation to the peak height offset calculated by the sum of the responses to each ligand individually. (A) Expected synergy ratio as a function of UDP and C5a dose (truncated at 1.5). The simulations show a ridge of synergy at a moderate UDP dose for most C5a doses. The black circles indicate dose combinations points of experiments that were conducted to test the model. (B) Expected synergy ratio as a function of UDP and C5a dose for a simulated GRK2 knockdown cell line. Without the GRK-mediated negative feedback to keep the IP3 generation from the C5a receptor within the non-linear range of calcium release the ridge in the synergy dose response is diminished. The synergy in the GRK knockdown simulation is not entirely eliminated because the shRNAi knockdown of GRK does not constitute a complete loss-of-function and low concentrations of ligand are still able to synergize. Furthermore, the asymmetric synergy dose response surface is more symmetric in the GRK knockdown simulation because the asymmetric calcium-dependent feedback mechanism is reduced.

The left panel of Figure 5 shows the results of model simulations at nominal parameters for a grid of doses of C5a and UDP. In the dose response surface, there is a ridge of synergistic calcium release for a moderate dose of UDP. We tested the model prediction with the experiment design measuring the synergy ratio at the points denoted as black open circles in the left panel of Figure 5. A χ^2^ goodness-of fit test comparing the model expected synergy ratio to the observed synergy ratio fails to reject the null hypothesis that the data were generated by the model mechanism (*p*-value≈1.0). The root-mean-squared error (RMSE) deviation between the predicted and actual experimental data is 0.492. By way of comparison, the RMSE between the data and the null model of no synergy is 1.044. We therefore conclude that the model predictions are consistent with the experimental observations. It should be noted that measurements of synergy in RAW cells are noisy and the ridge occurs at low doses of UDP. Notwithstanding, the phenomenon has been reported [40] and has been observed by us in this cell line.

The right panel of Figure 5 shows the same synergy dose response surface but for a GRK knockdown cell line. The synergy ridge observed in the wild-type cell simulation is changed in the GRK knockdown simulation indicating the C5a receptor desensitization mechanism mediated by GRK is important for the synergistic release of calcium. In the next section we pursue this conclusion in more detail, developing a conceptual explanation of the mechanism of crosstalk and synergy within our model.

## Discussion

G-protein-coupled receptors form a complex network of interacting proteins that generally exhibits the properties of a system in which each receptor signal is buffered from the others. For a minority of ligand combinations, however, crosstalk between pairs of receptors is apparent. Due to the complexity and importance of the system many hypothetical mechanisms have been proposed to explain the crosstalk [2]. In particular, simultaneous Gβγ and Gαq binding to PLCβ [20] and Gβγ exchange between Gαi and Gαq-coupled receptors have been proposed as potential mechanisms [19]. While our model does not eliminate these potential mechanisms, we do show that the mechanism represented in our model is consistent with a full range of experimental data including a variety of doses of C5a and UDP, C5a and UDP stimulation of five different knockdown cell-lines and double-ligand dose response experiments.

To our knowledge, this is the first multireceptor GPCR model and the first to address the complex phenomenon of crosstalk between GPCR receptor pathways that has been statistically estimated and validated with experimental data. This important phenomenon plays a role in processes as diverse as chemotaxis and perhaps drug interactions. In our model, the primary mechanism of synergy is due to the cooperative opening of the IP3 receptor. The robustness of the synergy is due to the feedback of GRK on the C5a receptor and the specificity of the synergy is due to the interaction patterns between specific Gα isoforms and PLCβ isoforms. The simultaneous binding model [20] accounts for the specificity of synergy, but not the robustness pattern of the synergy.

We observe in the model that if the Gαq-PLCβ3-Ca^2+^ and Gαq-PLCβ4-Ca^2+^ binding reactions are inhibited, the system still exhibits synergy. We conclude from this observation that the crosstalk mechanism is mediated by Gβγ. If the binding reaction of Gβγ to phosphorylated GRK2 is removed, the synergy is eliminated. Furthermore, if the GRK2-mediated phosphorylation of complexed C5a receptors is removed, the double ligand response is additive. We deduce then that the synergy mechanism involves GRK2 phosphorylation of complexed C5a receptors. However, GRK2 phosphorylation does not entirely explain the synergy mechanism.

In our model, the calcium released from the IP3 receptor is a function of the number of receptor molecules complexed to IP3 raised to the fourth power [41]. Therefore, for a small range of IP3 concentration, the amount of Ca^2+^ released is more than additive (see Figure S8). We conclude from our analysis of the model that the synergy ridge in Figure 5 arises because the GRK2 mediated mechanism holds the IP3 concentration in this non-additive region for most concentrations of C5a. The UDP response does not have the GRK2 mediated feedback and thus only shows a synergistic response for a small range of UDP concentration. If the GRK2 desensitization is removed from the model, the synergy ridge is removed and synergy is only present at low doses of C5a and UDP (see Figure 5).

The Bayesian method we have used for this model has several advantages for the estimation of model parameters in complex mechanistic system models. We have used an informative prior to exclude negative rate constants from the permitted parameter space. We have also used the prior distribution to center our a priori expectations of the rate constant at values obtained from *in-vitro* and other biochemical experiments. The Bayesian update rule allowed us to estimate parameters with our best current dataset and then update those estimates as new data became available from the calcium assay. In this way, we were able to iteratively refine and recalibrate our model with the most recent data available during data collection period for this project. The posterior distribution provides not only an estimate of the rate constants, but the entire distribution, from which we can calculate highest posterior confidence intervals and posterior correlations between parameters. For example, the posterior correlation between the binding and unbinding rates for the UDP-P2Y receptor complex were highly correlated, but those two constants were uncorrelated with the corresponding rates for the C5a-C5a receptor complex reaction even though we imposed no correlations a priori. Finally, the algorithmic methods for collecting ensembles of samples from the posterior distribution have improved considerably in recent years in terms of speed and robustness

We have shown that the signal transduction system as it is represented by our model does not require simultaneous binding of Gαq and Gβγ to PLCβ3 to cause a synergistic Ca^2+^ response due to simultaneous stimulation by C5a and UDP. We have shown that our representative model is consistent with this experimental dataset in RAW264.7 macrophage cells, but we have not excluded all other potential mechanisms that may be absent or regulated differently in this cell line compared to other macrophage cell lines. Indeed there are a few examples of statistical discrepancies between the model and experiments in our dataset (Table S4). These differences are substrate for further experimentation and modeling. The purpose of our model is to provide a quantitative tool to aid in reasoning about such complex interacting systems so that meaningful experiments can be designed to explore and understand the biological mechanism.

## Materials and Methods

The model equations are given in Figure S7. The initial conditions and parameter values are in Table S1 and Table S2, respectively. All the data used in this work and a stand-alone implementation of the model is provided at http://genomics.lbl.gov/supplemental/flaherty-gpcr/. The model was simulated using CVODE [42] and the GNU Scientific Library. Further details on materials and methods are available in Dataset S1.

### Experimental Methods

Intracellular free calcium in cultured adherent RAW264.7 cells was measured in a 96-well plate format using the Ca^2+^-sensitive fluorescent dye Fura-2 [43],[44]. A Molecular Devices FLEXstation scanning fluorometer was used to measure fluorescence using a bottom read of a 96-well plate. Each well was sampled approximately every 4 seconds. The measurement protocol is described in AfCS experimental protocol ID #PP00000211 (available from http://www.signaling-gateway.org). The parameters in ligand concentration model were estimated using FITC solution in the FLEXstation scanning fluorometer as described in Molecular Devices Maxline Application Note #45 and in Protocol S1 (see also Figure S5).

### Statistical Inference

Twenty of the 84 parameters were chosen to be estimated from data based on relevance to the experimental hypothesis. Only those parameters that related to the knockdown experiments in the dataset were estimated and are denoted with a star in Table S2. We used data to estimate only the two forward rate constants in the enzymatic mass-action equations because the forward and reverse rate constants for a given reaction will be highly correlated in the posterior distribution making estimation by Markov chain methods computationally expensive. An analysis of the sensitivity of the model to each parameter is shown in Figure S9.

For each estimated parameter we constructed an independent Gaussian prior on a log scale with a mean chosen based on relevant literature and a standard deviation of 0.25. We found that this prior variance was sufficiently permissive to allow exploration of the space while still constraining the rates to be physically reasonable. The prior distribution over the parameters allows the incorporation of both soft and hard constraints in the parameter estimates. Parameter sets with zero measure are not permitted in the posterior distribution and parameter sets with small measure must be assigned a large likelihood in order to have a large posterior probability.

The likelihood is a function of the parameters (*θ*) and links the prior distribution with the posterior distribution under Bayes rule
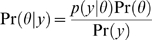
where *y* denotes the observed data.

In our model, the likelihood function is a Gaussian distribution according to the non-linear regression equation *y* = *f*(*θ*)+*ε*, *ε*∼*N*(0,*σ* ^2^), where *f*(*θ*) is the deterministic model prediction. The posterior distribution is of interest because it informs us as to the most probable setting of the parameters as well as the uncertainty in the values.

The Metropolis-Hastings algorithm [45] was used to estimate the posterior density of the parameters Pr(*θ*|*y*). Three independent chains were simulated from different initial parameter values (see Figure S1). To assess convergence of the posterior distribution estimate, we used the Gelman-Rubin potential scale reduction factor (PSRF) [46]. The multivariate PSRF is 2.44 and 95% of the individual PSRFs were less than 1.5. A PSRF value of one indicates that the distribution has converged and values near one are close to converged.

Posterior prediction confidence intervals were constructed using the percentiles from the predictive distribution approximated with 2000 Monte Carlo samples from Pr(*y*_new_|*θ_i_*) at each of 100 simple random samples from Pr(*θ*|*y*) obtained from
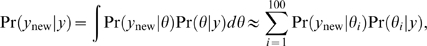
where Pr(*y*_new_|*θ_i_*)∼*N*(*f*(*θ*),*s* ^2^) and *s* ^2^ is the pooled variance estimate, which is computed as an average of the variances of all the time points in each of the 29 wild-type experiments. These average variances were weighted by the number of technical replicates in each experiment and then averaged to yield the estimate *s* ^2^. A small factor of 1 nM^2^ was added to each variance estimate to bound variance estimates away from zero.

## Supporting Information

Dataset S1 (0.11 MB DOC)

Figure S1 This figure shows exemplar MCMC realizations for parameter k109f (the UDP+P2YR forward binding rate) from three independent chains. The chains have converged to the stationary distribution which is the posterior distribution as measured by the PSRF (see Materials and Methods).
(0.20 MB DOC)

Figure S2 Posterior distributions and correlations The first figure shows that the pairwise marginal posterior distributions for the ligand binding reactions for P2YR and C5aR. The posterior distributions show the dissociation constants for the reactions are tightly constrained by the data, while the values of the forward and reverse rates that make up the ratio are not as well constrained by the data. Additionally, the UDP binding rates are not correlated with the C5a binding rates. k108f and k108r are the P2YR forward and reverse rates and k101f and k101r are the C5aR rates. The next two figures show the one-way marginal posterior density estimates from three independent MCMC chains with approximately 30,000 samples. The 20 estimates parameters are along the rows and the independent chains are along the columns. In each plot, the light blue density is the prior density and the green, purple and orange densities are the posterior densities. The vertical line shows the parameter value used in the model simulations in the paper and listed in Table S3. All of the densities are plotted on a log scale. Each marginal posterior distribution estimate is constructed from independent MCMC chains. The results from each chain (three of them) are shown in the columns of the second figure below. In some cases the algorithm sampled heavily from one mode that was not explored as heavily by another chain. However, the PSRF criterion used to assay convergence and a visual inspection of overall posterior density correspondence do indicate that the posterior distributions are sufficiently sampled by all three chains in aggregate. Furthermore, the fit of the model to the data as shown in Figure S3 shows that the model point estimates are effective in fitting the actual calcium measurements.
(0.55 MB DOC)

Figure S3 Peak height dose response. This figure shows the single ligand calcium dose responses for C5a and UDP stimulation.
(0.21 MB DOC)

Figure S4 Knockdown simulations. This figure shows representative simulations and data for each knockdown experiment. A complete set of all 96 experiments is provided in a supplementary folder.
(0.69 MB DOC)

Figure S5 Input model fit. This figure shows the input model (described in Materials and Methods) fit to the FITC measurements. The ligand concentration that the cell sees does not transit instantaneously from 0 to the final concentration. The ligand concentration is expected to take an amount of time that is significant on the scale of the measurements made for this study to reach the final concentration.
(0.12 MB DOC)

Figure S6 Large pathway diagram.
(0.18 MB DOC)

Figure S7 System of differential equations. This figure shows the complete set of differential equations used to simulate the model. These equations are also available in the source c code for the model supplied. This system of equations with the initial conditions and nominal parameter values reported in Table S1 and Table S2, respectively, completely define the model and allow for the reproduction of the simulations used in this paper on any platform.
(1.62 MB DOC)

Figure S8Hill function self-synergy. Consider a Hill function, 
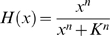
. 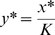 is a dimensionless critical concentration y*, below which self-synergy will occur. Based on the analysis, we conclude that: (i) *n* must be greater than 1 for self-synergy to occur, (ii) self synergy never occurs if the concentration *x* exceeds equilibrium constant *K* (*y*>1), and (iii) for *n*>2, there is a large range of concentration for self-synergy. In the G protein model, *x*, is the concentration of IP3-IP3R, *H*(*x*) is the rate of change in cytosolic calcium concentration and *n* = 4. We have tested the validity of this self synergy hypothesis by stimulating the cells with both 20 nM UDP and 40 nM UDP (data not shown). Though at such low ligand concentrations, the measurement variability is high, we observed that the synergy ratio, on average was 1.17 compared to a value of 1.25 predicted by the model.(0.02 MB DOC)

Figure S9 Parameter sensitivity analysis. The parameter of interest is varied by 10% while all other parameters are kept constant. The parameters are grouped according to their functionalities. The sensitivity coefficient is the ratio of the relative change in the peak height to the relative change in the parameter value. The four most sensitive parameters (sensitivity coefficient >2) in the Cacyt category are Vqssk50 (IP3+IP3K_a->IP4+IP3K_a (Vmax)), Kqssk50 (IP3+IP3K_a->IP4+IP3K_a (Km)), a1 (Ca leak into the cell from outside), and Kex (Na/Ca exchange activation const). The top 3 most sensitive parameters in the PLCb3 category are: k21bf* (PLCb3_Ca_Gbg_PIP2->PLCb3_Ca_Gbg+IP3+DAG), k20f (Gbg+PLCb3_Ca->PLCb3_Ca_Gbg), k21af* (PLCb3_Ca_Gbg+PIP2->PLCb3_Ca_Gbg_PIP2). A star next to the parameter name indicates it was estimated.
(0.04 MB DOC)

Protocol S1 FITC protocol.
(0.03 MB DOC)

Table S1 Model initial conditions. This table shows the initial conditions used for the model. The model was run for sufficient time for the species states in the model to reach equilibrium before ligand stimulation was added. The number of molecules was calculated using a cell volume of 1 pL.
(0.05 MB DOC)

Table S2 Model parameters. This table shows the nominal parameters used for the model. Parameter distributions that were estimated are shown as shaded rows and with a star next to the parameter name in the table. The prior distribution for each parameter is as described in the Materials and Methods section with mean value specified by the column labeled “prior”.
(0.14 MB DOC)

Table S3 Parameter posterior uncertainty and references. This table shows the HPD intervals as computed by the R CODA library function “hpdinterval”. HPD intervals for each of the three MCMC chains were calculated and the union of those intervals is reported for each parameter in this table. The prior value reported in Table S2 was set using information from references listed in the appropriate column. The references used to form the basis of the parameter estimates are shown in the last column.
(0.37 MB DOC)

Table S4 Goodness of fit evaluation. We use the mean squared error criterion to evaluate the goodness of our model fit to the data. We have used this data in the estimation procedure and thus does not constitute a true validation. However, we show that in general our model fits the bulk of the data. Those areas of lack-of-fit are usually due to extraordinary experiment-to-experiment variation and in some cases point to unaccounted mechanisms. We elaborate on one such mechanism (multiple GRK isoforms) in the text of the article.
(0.27 MB DOC)

## References

[1] Kroeze WK, Sheffler DJ, Roth BL (2003). G-protein-coupled receptors at a glance.. J Cell Sci.

[2] Werry TD, Wilkinson GF, Willars GB (2003). Mechanisms of cross-talk between G-protein-coupled receptors resulting in enhanced release of intracellular Ca^2+^.. Biochem J.

[3] Casey PJ, Gilman AG (1988). G protein involvement in receptor-effector coupling.. J Biol Chem.

[4] Wu D, Katz A, Simon MI (1993). Activation of phospholipase C β_2_ by the α and βγ subunits of trimeric GTP-binding protein.. Proc Natl Acad Sci U S A.

[5] Mukhopadhyay S, Ross EM (1999). Rapid GTP binding and hydrolysis by G_q_ promoted by receptor and GTPase-activating proteins.. Proc Natl Acad Sci U S A.

[6] Patterson RL, Boehning D, Snyder SH (2004). Inositol 1,4,5-trisphosphate receptors as signal integrators.. Annu Rev Biochem.

[7] Ananthanarayanan B, Stahelin RV, Digman MA, Cho W (2003). Activation mechanisms of conventional protein kinase C isoforms are determined by the ligand affinity and conformational flexibility of their C1 domains.. J Biol Chem.

[8] Penela P, Ribas C, Mayor F (2003). Mechanisms of regulation of the expression and function of G protein-coupled receptor kinases.. Cell Signal.

[9] Pitcher JA, Touhara K, Payne ES, Lefkowitz RJ (1995). Pleckstrin homology domain-mediated membrane association and activation of the β-adrenergic receptor kinase requires coordinate interaction with G_βγ_ subunits and lipid.. J Biol Chem.

[10] Berg JM, Tymoczko JL, Stryer L (2002). Biochemistry. 5th ed.

[11] Allegretti M, Moriconi A, Beccari AR, Di Bitondo R, Bizzarri C (2005). Targeting C5a: recent advances in drug discovery.. Curr Med Chem.

[12] Warny M, Aboudola S, Robson SC, Sevigny J, Communi D (2001). P2Y_6_ nucleotide receptor mediates monocyte interleukin-8 production in response to UDP or lipopolysaccharide.. J Biol Chem.

[13] Yoshioka K, Saitoh O, Nakata H (2001). Heteromeric association creates a P2Y-like adenosine receptor.. Proc Natl Acad Sci U S A.

[14] Lukas TJ (2004). A signal transduction pathway model prototype I: from agonist to cellular endpoint.. Biophys J.

[15] Mishra J, Bhalla US (2002). Simulations of inositol phosphate metabolism and its interaction with InsP_3_-mediated calcium release.. Biophys J.

[16] Lemon G, Gibson WG, Bennett MR (2003). Metabotropic receptor activation, desensitization and sequestration—I: modelling calcium and inositol 1,4,5-trisphosphate dynamics following receptor activation.. J Theor Biol.

[17] Maurya MR, Subramaniam S (2007). A kinetic model for calcium dynamics in RAW 264.7 cells: 2. Knockdown response and long-term response.. Biophys J.

[18] Maurya MR, Subramaniam S (2007). A kinetic model for calcium dynamics in RAW 264.7 Cells: 1. Mechanisms, parameters and sub-populational variability.. Biophys J.

[19] Quitterer U, Lohse MJ (1999). Crosstalk between Gα_i_- and Gα_q_-coupled receptors is mediated by Gβγ exchange.. Proc Natl Acad Sci U S A.

[20] Zhu X, Birnbaumer L (1996). G protein subunits and the stimulation of phospholipase C by G_s_- and G_i_-coupled receptors: lack of receptor selectivity of Gα_16_ and evidence for a synergic interaction between Gβγ and the α subunit of a receptor activated G protein.. Proc Natl Acad Sci U S A.

[21] Kehrl JH (1998). Heterotrimeric G protein signaling: roles in immune function and fine-tuning by RGS proteins.. Immunity.

[22] Ross EM, Wilkie TM (2000). GTPase-activating proteins for heterotrimeric G proteins: regulators of G protein signaling (RGS) and RGS-like proteins.. Annu Rev Biochem.

[23] Cunningham ML, Waldo GL, Hollinger S, Hepler JR, Harden TK (2001). Protein kinase C phosphorylates RGS2 and modulates its capacity for negative regulation of Gα_11_ signaling.. J Biol Chem.

[24] Kehrl JH, Sinnarajah S (2002). RGS2: a multifunctional regulator of G-protein signaling.. Int J Biochem Cell Biol.

[25] Keizer J, De Young GW (1992). Two roles of Ca^2+^ in agonist stimulated Ca^2+^ oscillations.. Biophys J.

[26] Meyer T, Holowka D, Stryer L (1988). Highly cooperative opening of calcium channels by inositol 1,4,5-trisphosphate.. Science.

[27] Meyer T, Stryer L (1988). Molecular model for receptor-stimulated calcium spiking.. Proc Natl Acad Sci U S A.

[28] Jiang H, Kuang Y, Wu Y, Smrcka A, Simon MI (1996). Pertussis toxin-sensitive activation of phospholipase C by the C5a and fMet-Leu-Phe receptors.. J Biol Chem.

[29] Park D, Jhon DY, Lee CW, Lee KH, Rhee SG (1993). Activation of phospholipase C isozymes by G protein βγ subunits.. J Biol Chem.

[30] Smrcka AV, Sternweis PC (1993). Regulation of purified subtypes of phosphatidylinositol-specific phospholipase C β by G protein α and βγ subunits.. J Biol Chem.

[31] Lee CW, Lee KH, Lee SB, Park D, Rhee SG (1994). Regulation of phospholipase C-β4 by ribonucleotides and the α subunit of G_q_.. J Biol Chem.

[32] Jiang H, Wu D, Simon MI (1994). Activation of phospholipase C β4 by heterotrimeric GTP-binding proteins.. J Biol Chem.

[33] Runnels LW, Scarlata SF (1999). Determination of the affinities between heterotrimeric G protein subunits and their phospholipase C-β effectors.. Biochemistry.

[34] Rhee SG (2001). Regulation of phosphoinositide-specific phospholipase C.. Annu Rev Biochem.

[35] Yue C, Ku CY, Liu M, Simon MI, Sanborn BM (2000). Molecular mechanism of the inhibition of phospholipase C β_3_ by protein kinase C.. J Biol Chem.

[36] Litosch I (2002). Novel mechanisms for feedback regulation of phospholipase C-β activity.. IUBMB Life.

[37] Spitaler M, Cantrell DA (2004). Protein kinase C and beyond.. Nat Immunol.

[38] Langkabel P, Zwirner J, Oppermann M (1999). Ligand-induced phosphorylation of anaphylatoxin receptors C3aR and C5aR is mediated by G protein-coupled receptor kinases.. Eur J Immunol.

[39] Shin KJ, Wall EA, Zavzavadjian JR, Santat LA, Liu J (2006). A single lentiviral vector platform for microRNA-based conditional RNA interference and coordinated transgene expression.. Proc Natl Acad Sci U S A.

[40] Natarajan M, Lin KM, Hsueh RC, Sternweis PC, Ranganathan R (2006). A global analysis of cross-talk in a mammalian cellular signalling network.. Nat Cell Biol.

[41] De Young GW, Keizer J (1992). A single-pool inositol 1,4,5-trisphosphate-receptor-based model for agonist-stimulated oscillations in Ca^2+^ concentration.. Proc Natl Acad Sci U S A.

[42] Hindmarsh AC, Brown PN, Grant KE, Lee SL, Serban R (2005). SUNDIALS: Suite of Nonlinear and Differential/Algebraic Equation Solvers.. ACM Trans Math Softw.

[43] Tsien RY (1989). Fluorescent indicators of ion concentrations.. Methods Cell Biol.

[44] Grynkiewicz G, Poenie M, Tsien RY (1985). A new generation of Ca^2+^ indicators with greatly improved fluorescence properties.. J Biol Chem.

[45] Robert CP, Casella G (2004). Monte Carlo Statistical Methods.

[46] Gelman A, Rubin DB (1992). Inference from iterative simulation using multiple sequences.. Stat Sci.

