# Supplementary material for: A Dual Receptor Crosstalk Model of G-Protein-Coupled Signal Transduction
Source: PLoS Comput Biol. 2008 Sep 26;4(9):e1000185. doi: 10.1371/journal.pcbi.1000185 (PMC2528964; doi:10.1371/journal.pcbi.1000185)
Supplement: Dataset S1 — (0.11 MB DOC) [file pcbi.1000185.s001.doc]

### Data Set

The time interval between samples is approximately 3-4 seconds and each time series is approximately 100-300 seconds of post-stimulation data. Table 1 shows a summary of the knockdown data used for statistical parameter estimation for this model in addition to the wild-type experiments.

|  |  |  | |  |  |  | **Sample Size** | | | | | |
| --- | --- | --- | --- | --- | --- | --- | --- | --- | --- | --- | --- | --- |
|  | **Measured Fraction Knockdown** | | | **Model Value** | | | **C5a** | | | **UDP** | | |
| Cell Line | qRT-PCR | | Western | Nominal | Lower | Upper | <10nM | 10 - 100nM | >100nM | <1μM | 1 –  10μM | > 10μM |
| Wild-type | - | | - | - | - | - | 4 | 8 | 3 | 5 | 5 | 4 |
| GRK2 (2) | 90% ± 7%, n=5 | | 40% ± 6%, n=6 | 40.0% | 22.0% | 58.0% | 2 | 12 | 2 | 3 | 1 | 5 |
| Gai2 (3) | 83% ± 5%, n=4 | | 73% ± 6%, n=5 | 73.0% | 55.0% | 91.0% | - | 5 | - | 5 | - | 7 |
| Gaq (3) | 70% ± 8%, n=7 | | 66% ± 23%, n=2 | 66.0% | 0.0% | 95.0% | - | 3 | - | 1 | - | 3 |
| PLCb3 (1) | - | | 83% ± 15%, n=3 | 83.0% | 38.0% | 100.0% | - | 3 | - | - | - | 3 |
| PLCb4 (1) | 87% ± 6%, n=5 | | - | 87.0% | 69.0% | 100.0% | - | 4 | - | 4 | - | 4 |

Table 1: The data set used for parameter estimation is shown in this table. Five different cell lines which have a perturbation in the level of a key signal transduction protein were constructed by shRNAi lentiviral infection. The calcium response from these cell lines in addition to the wild-type cell line were used to fit relevant parameters in the model. Since shRNAi does not entirely remove the protein product, the fraction knockdown was estimated by qRT-PCR and by Western blot analysis. The standard error (se) was computed for each estimate and the upper and lower confidence intervals were computed as ±3·se. The knockdown confidence intervals are used in the GPCR model to construct prediction confidence intervals for the calcium response. Where several cell lines were constructed for each knockdown, the best was selected and reported in parenthesis. The sample size for each knockdown-ligand dose combination is shown in the last 6 columns.

This data set was first used to check the accuracy of the model. The five knockdown perturbations and the range of ligand doses impose strong quantitative constraints on the model.

### Model & Data Preprocessing

From the vantage point of an average cell, the concentration of ligand is at first zero, then as the ligand molecules diffuse in the media the effective concentration at the membrane interface asymptotes to the equilibrium concentration. We employ the following model for the ligand concentration at the plasma membrane interface as a function of time

$$y(t)=\left\{\begin{matrix} \frac{a_{1}(t-t_{0})}{(t-t_{0})+a_{2}}+a_{3} & t>t_{0} \\ a_{3} & t\leq t_{0} \end{matrix}\right.$$

and fit parameters of the model using FITC measurements as described in the supplementary information. Figure S5 shows the fit of the model to the FITC data.

Fura-2 measurements report only the relative change in cytosolic calcium and are not able to report absolute calcium levels. In order that the baseline concentration of cytosolic calcium in our model match a reasonable resting cytosolic calcium concentration we subtracted the average the prestimulus calcium concentration for each experiment and recentered the baseline at 80nM which is a reasonable physiological level for these cells.

In our model we have lumped PLCβ2 and PLCβ3 because their regulators and effectors in the context of the rest of the calcium model are identical. However, the experimental perturbations of PLCβ3 left PLCβ2 concentrations unchanged. Accordingly, we took the knockdown fraction to be 50% of that reported by western blot analysis for the purposes of simulation of PLCβ3 knockdown experiments.

## Detailed Statistical Inference

Twenty of the 84 parameters were chosen to be estimated from data based on relevance to the experimental data. Only those parameters that related to the knockdown experiments in the data set were estimated and are denoted with a star in Table S2. We used data to estimate only the two forward rate constants in the enzymatic mass-action equations because the forward and reverse rate constants for a given reaction will be highly correlated in the posterior distribution making estimation by Markov chain methods computationally expensive.

For each estimated parameter we constructed an independent Gaussian prior on a log scale with a mean chosen based on relevant literature and a standard deviation of 0.25. We found that this prior variance was sufficiently permissive to the exploration of the space while still constraining the rates to be physically reasonable. The prior distribution over the parameters allows the incorporation of both soft and hard constraints in the parameter estimates. Parameter sets with zero measure are not permitted in the posterior distribution and parameter sets with small measure must be assigned a large likelihood in order to have a large posterior probability.

The likelihood function links the prior distribution with the posterior distribution under Bayes rule

$\Pr(\theta|y)=\frac{p(y|\theta)\Pr(\theta)}{\Pr(y)}$.

In our model, the likelihood function is a Gaussian distribution according to the non-linear regression equation, where $f(\theta)$ is the deterministic model prediction. The posterior distribution is of interest because it informs us as to the most probable setting of the parameters as well as the uncertainty in the values.

The Metropolis-Hastings algorithm (1) was used to estimate the posterior density of the parameters Pr(θ|**y**) . Since the posterior density of the parameters has significant correlation structure, three independent chains were simulated from different initial parameter values. Each chain was simulated for a burn-in period of 50,000 iterations and then a sample size of 29906 was taken with a thinning factor of 10. To assess convergence of the posterior distribution estimate, we used the Gelman-Rubin potential scale reduction factor (PSRF) (2). The multivariate PSRF is 2.44 and 95% of the individual PSRFs were less than 1.5. A PSRF value of one indicates that the distribution has converged and values near one are close to converged.

Posterior prediction confidence intervals were constructed using the percentiles from the predictive distribution approximated with 2000 Monte Carlo samples from $\Pr(y_{\text{new}}|\theta_{i})$ at each of 100 simple random samples from $\Pr(\theta|y)$ according to

where and $\hat{s}^{2}$ is the pooled variance estimate, which is computed as an average of the variances of all the time points in each of the 29 wild-type experiments. These average variances are weighted by the number of technical replicates in each experiment and then averaged to yield the estimate $\hat{s}^{2}$.

The observed standard deviation for each calcium measurement was obtained from 3-4 replicates on the same plate. By chance the replicate measurements for some time points were nearly identical causing the standard deviation estimate to be close to zero. Since the log of the likelihood for a Gaussian distribution contains the standard deviation estimate in the denominator, a near-zero value will force the likelihood to be very large unless a parameter value is selected which causes the simulation value to be very close to the measured value in the numerator. This effectively causes only a few terms in the likelihood to have a disproportionate importance in the model fit. We implemented a common remedy for this situation. A small constant factor (1nM) was added to the estimate of the standard deviation.

# References
