# Supplementary material for: A Dual Receptor Crosstalk Model of G-Protein-Coupled Signal Transduction
Source: PLoS Comput Biol. 2008 Sep 26;4(9):e1000185. doi: 10.1371/journal.pcbi.1000185 (PMC2528964; doi:10.1371/journal.pcbi.1000185)

Figure S1:

This figure shows exemplar MCMC realizations for parameter k109f (the UDP+P2YR forward binding rate) from three independent chains. The chains have converged to the stationary distribution which is the posterior distribution as measured by the PSRF (see Materials and Methods).


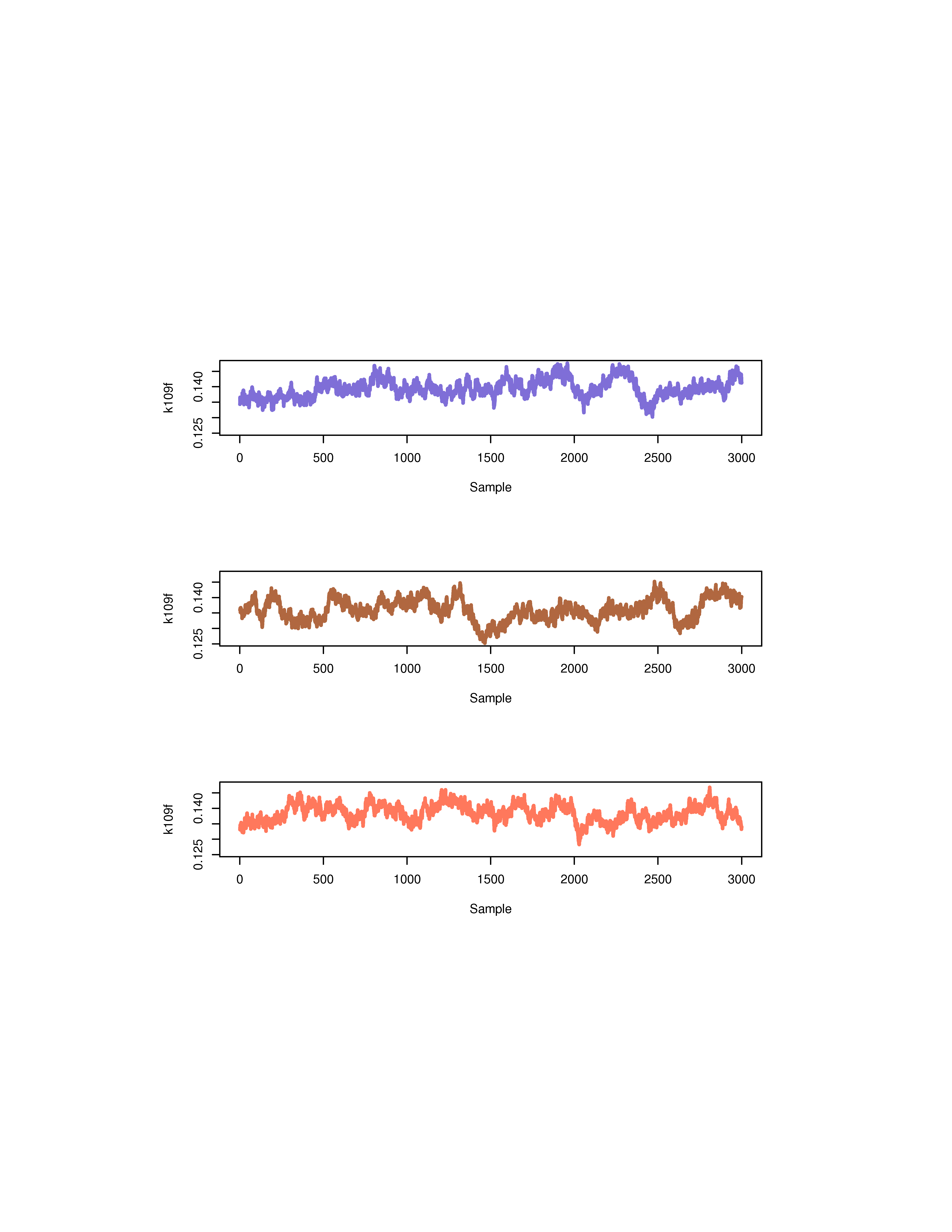

Supplement: Figure S1 — This figure shows exemplar MCMC realizations for parameter k109f (the UDP+P2YR forward binding rate) from three independent chains. The chains have converged to the stationary distribution which is the posterior distribution as measured by the PSRF (see Materials and Methods). (0.20 MB DOC) [file pcbi.1000185.s002.doc]
