# Supplementary material for: A Dual Receptor Crosstalk Model of G-Protein-Coupled Signal Transduction
Source: PLoS Comput Biol. 2008 Sep 26;4(9):e1000185. doi: 10.1371/journal.pcbi.1000185 (PMC2528964; doi:10.1371/journal.pcbi.1000185)

Figure S2: Posterior Distributions & Correlations

The first figure shows that the pairwise marginal posterior distributions for the ligand binding reactions for P2YR and C5aR. The posterior distributions show the dissociation constants for the reactions are tightly constrained by the data, while the values of the forward and reverse rates that make up the ratio are not as well constrained by the data. Additionally, the UDP binding rates are not correlated with the C5a binding rates. k108f and k108r are the P2YR forward and reverse rates and k101f and k101r are the C5aR rates.

The next two figures show the one-way marginal posterior density estimates from three independent MCMC chains with approximately 30,000 samples. The 20 estimates parameters are along the rows and the independent chains are along the columns. In each plot, the light blue density is the prior density and the green, purple and orange densities are the posterior densities. The vertical line shows the parameter value used in the model simulations in the paper and listed in table S3. All of the densities are plotted on a log scale.

Each marginal posterior distribution estimate is constructed from independent MCMC chains. The results from each chain (three of them) are shown in the columns of the second figure below. In some cases the algorithm sampled heavily from one mode that was not explored as heavily by another chain. However, the PSRF criterion used to assay convergence and a visual inspection of overall posterior density correspondence do indicate that the posterior distributions are sufficiently sampled by all three chains in aggregate. Furthermore, the fit of the model to the data as shown in figure S3 shows that the model point estimates are effective in fitting the actual calcium measurements.


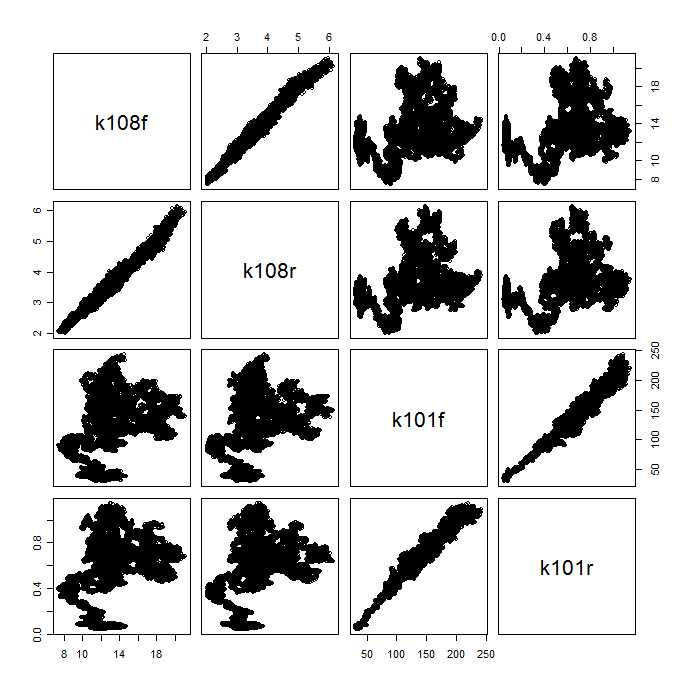


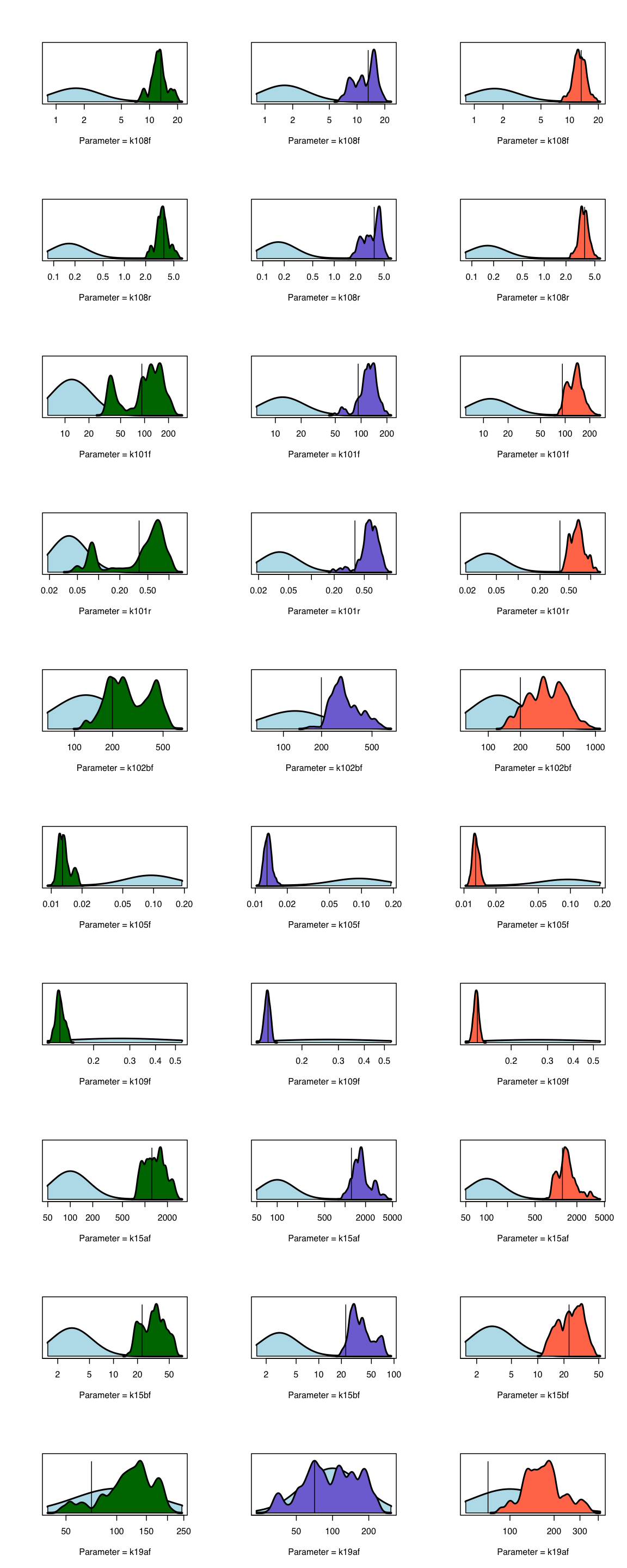


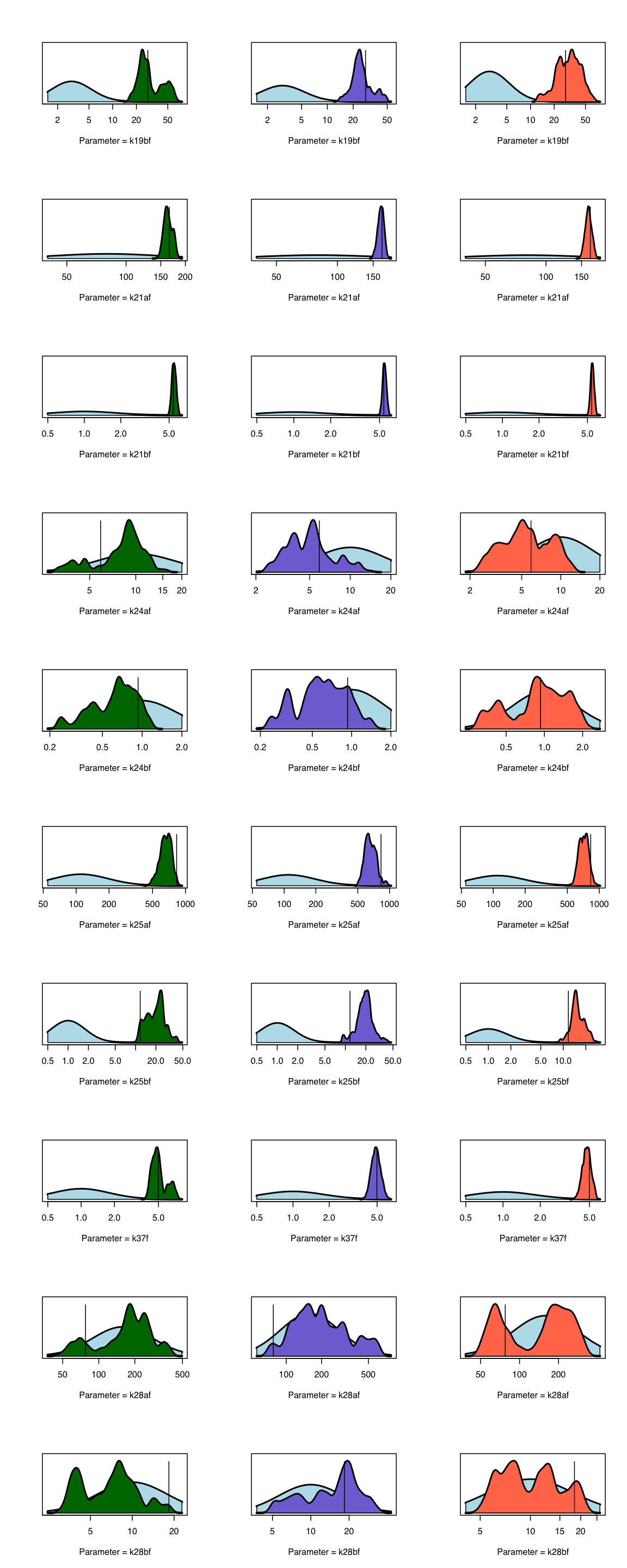

Supplement: Figure S2 — Posterior distributions and correlations The first figure shows that the pairwise marginal posterior distributions for the ligand binding reactions for P2YR and C5aR. The posterior distributions show the dissociation constants for the reactions are tightly constrained by the data, while the values of the forward and reverse rates that make up the ratio are not as well constrained by the data. Additionally, the UDP binding rates are not correlated with the C5a binding rates. k108f and k108r are the P2YR forward and reverse rates and k101f and k101r are the C5aR rates. The next two figures show the one-way marginal posterior density estimates from three independent MCMC chains with approximately 30,000 samples. The 20 estimates parameters are along the rows and the independent chains are along the columns. In each plot, the light blue density is the prior density and the green, purple and orange densities are the posterior densities. The vertical line shows the parameter value used in the model simulations in the paper and listed in Table S3. All of the densities are plotted on a log scale. Each marginal posterior distribution estimate is constructed from independent MCMC chains. The results from each chain (three of them) are shown in the columns of the second figure below. In some cases the algorithm sampled heavily from one mode that was not explored as heavily by another chain. However, the PSRF criterion used to assay convergence and a visual inspection of overall posterior density correspondence do indicate that the posterior distributions are sufficiently sampled by all three chains in aggregate. Furthermore, the fit of the model to the data as shown in Figure S3 shows that the model point estimates are effective in fitting the actual calcium measurements. (0.55 MB DOC) [file pcbi.1000185.s003.doc]
