# Supplementary material for: A Dual Receptor Crosstalk Model of G-Protein-Coupled Signal Transduction
Source: PLoS Comput Biol. 2008 Sep 26;4(9):e1000185. doi: 10.1371/journal.pcbi.1000185 (PMC2528964; doi:10.1371/journal.pcbi.1000185)

Figure S3: Peak Height Dose Response

This figure shows the single ligand calcium dose responses for C5a and UDP stimulation.


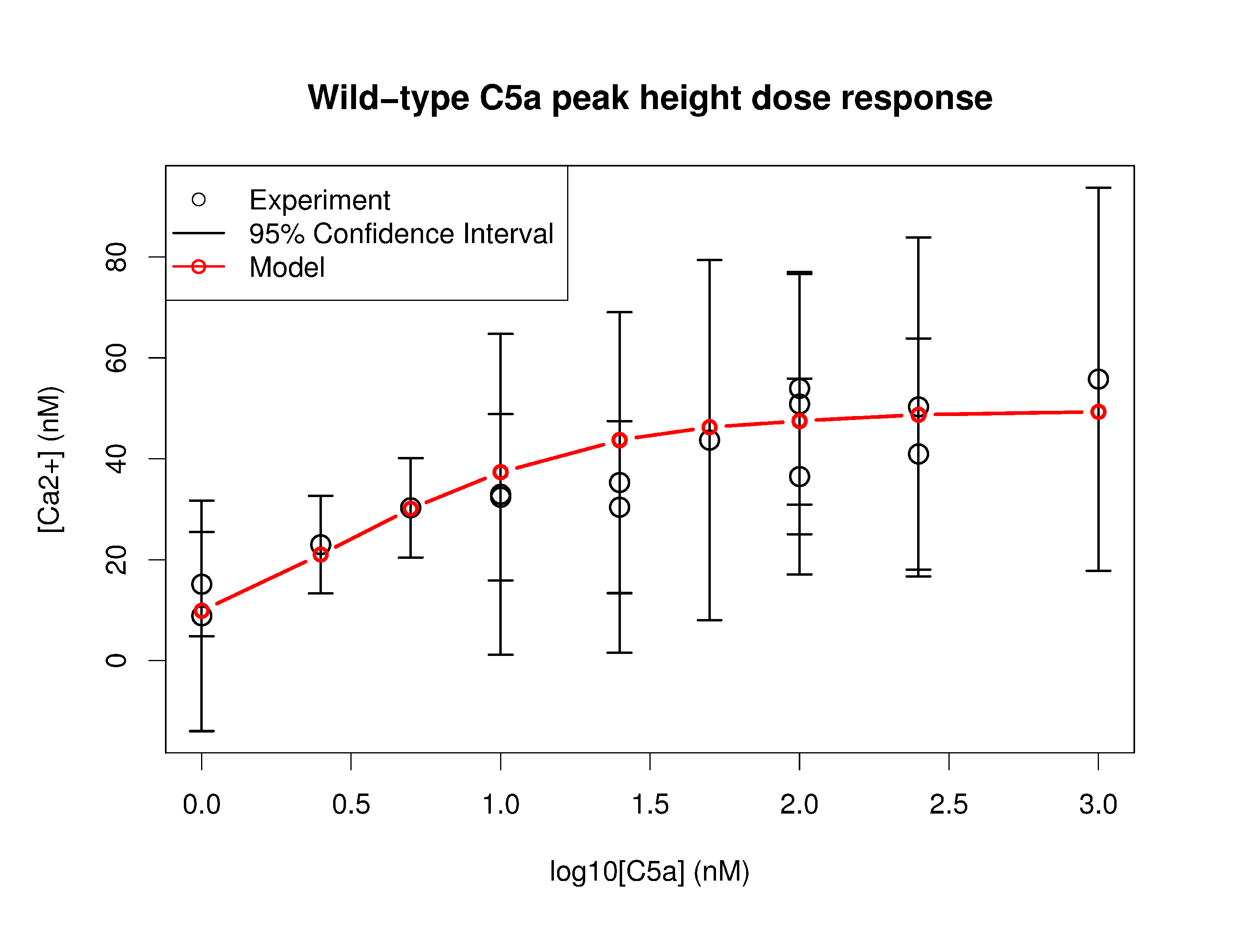


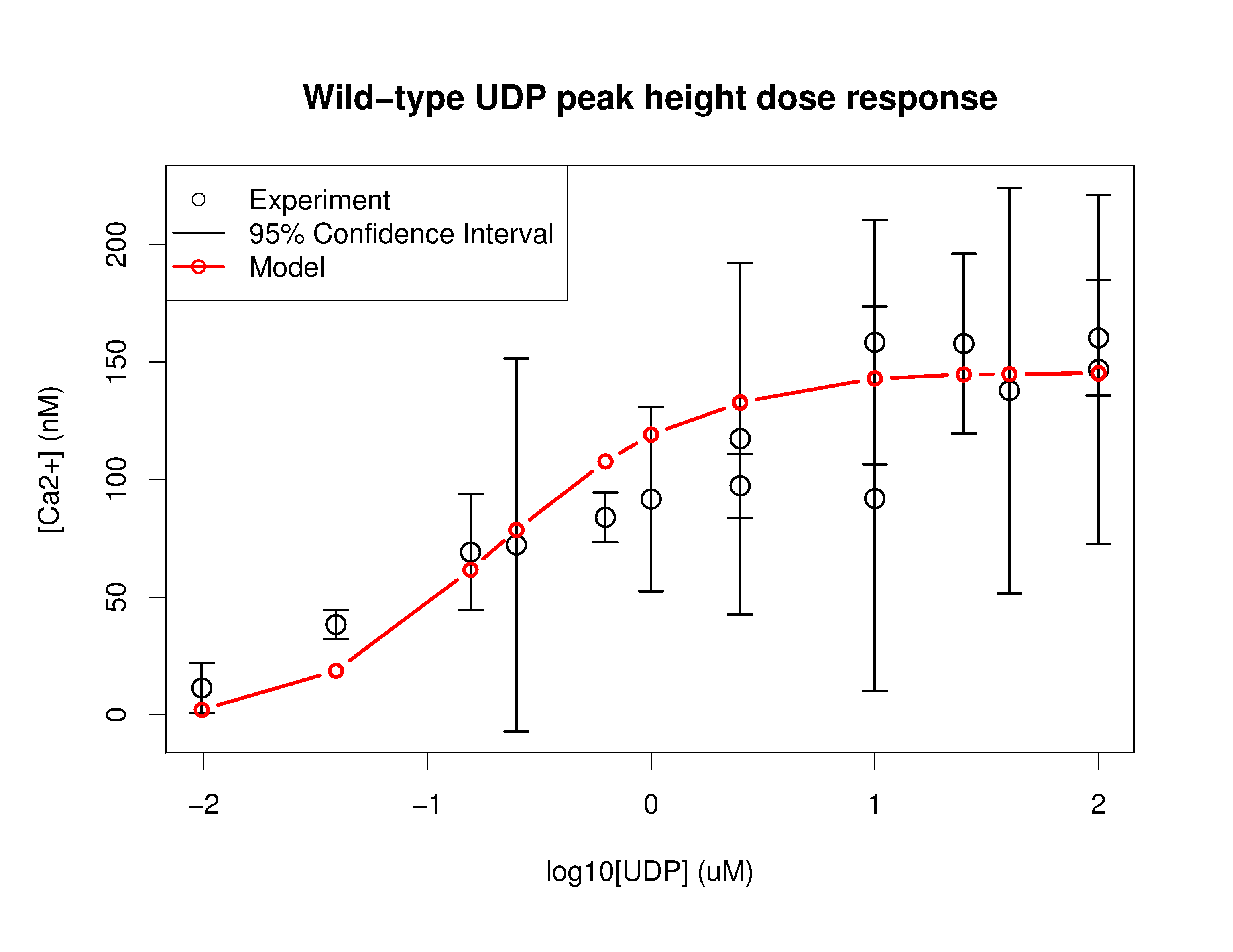

Supplement: Figure S3 — Peak height dose response. This figure shows the single ligand calcium dose responses for C5a and UDP stimulation. (0.21 MB DOC) [file pcbi.1000185.s004.doc]
