# Supplementary material for: A Dual Receptor Crosstalk Model of G-Protein-Coupled Signal Transduction
Source: PLoS Comput Biol. 2008 Sep 26;4(9):e1000185. doi: 10.1371/journal.pcbi.1000185 (PMC2528964; doi:10.1371/journal.pcbi.1000185)
Supplement: Figure S4 — Knockdown simulations. This figure shows representative simulations and data for each knockdown experiment. A complete set of all 96 experiments is provided in a supplementary folder. (0.69 MB DOC) [file pcbi.1000185.s005.doc]

Figure S4: Knockdown Simulations

This figure shows representative simulations and data for each knockdown experiment. A complete set of all 96 experiments is provided in a supplementary folder.

|  | C5a | UDP |
| --- | --- | --- |
| Wild-type | 250nM  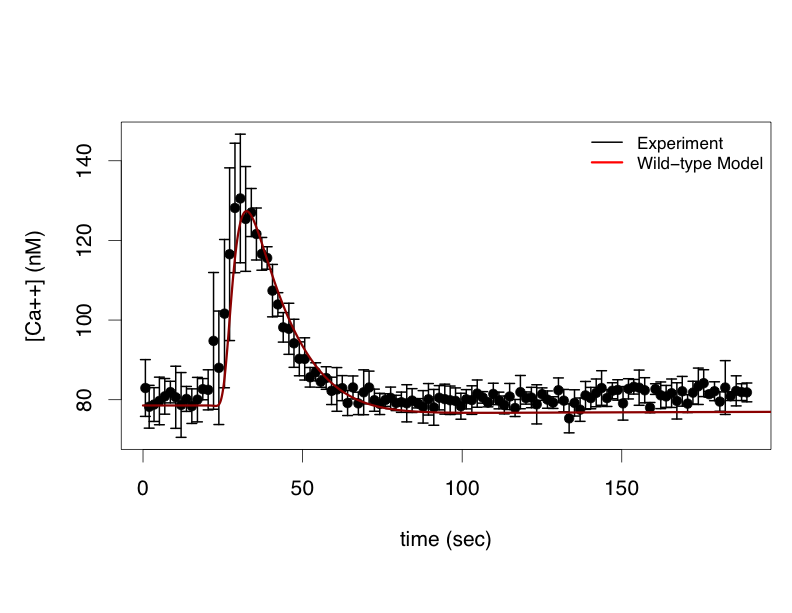 | 25μM  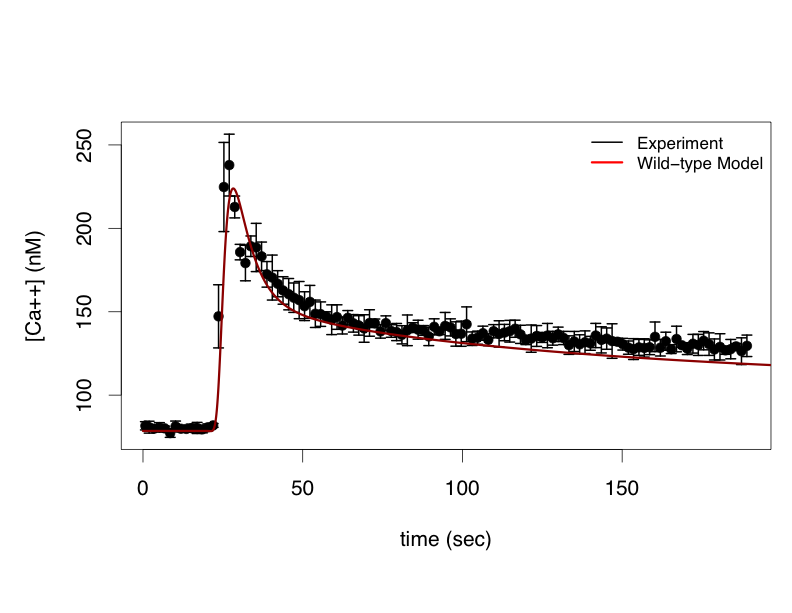 |
| GRK2 | 250nM  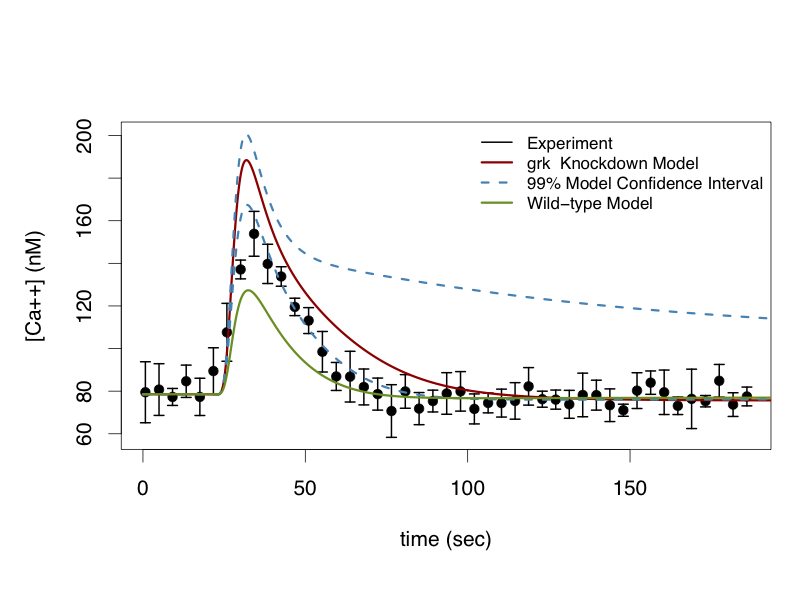 | 25μM  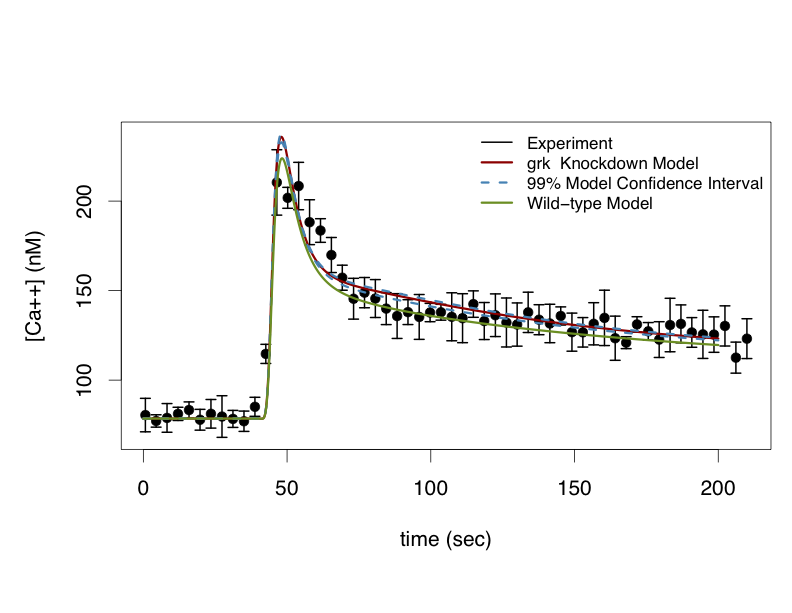 |
| Gαi2 | 100nM  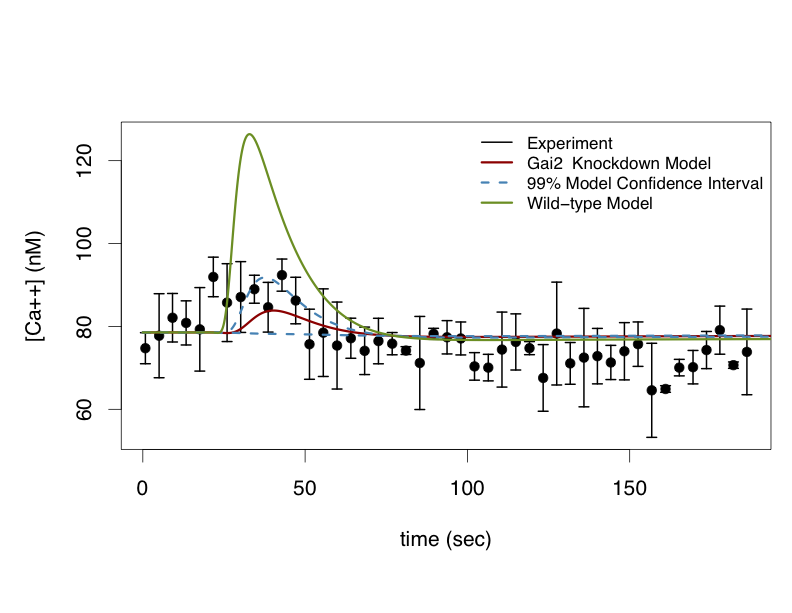 | 25μM  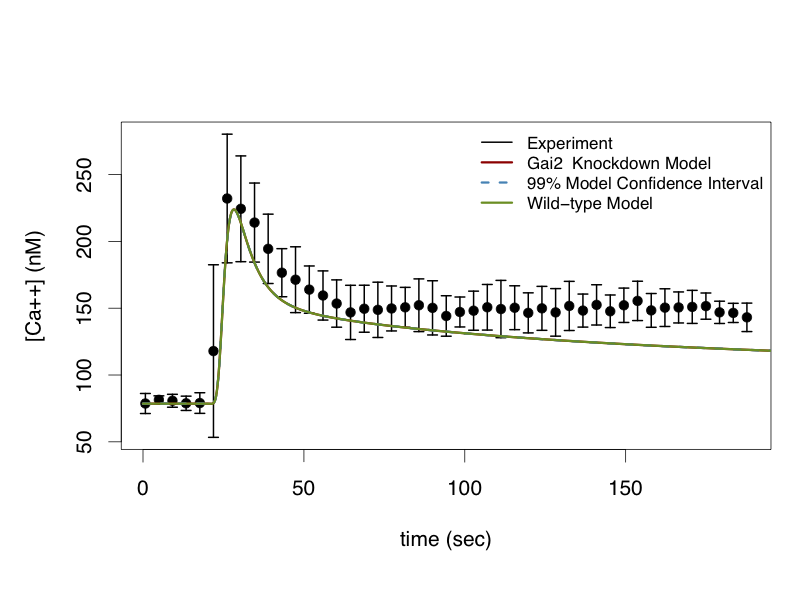 |
| Gαq | 100nM  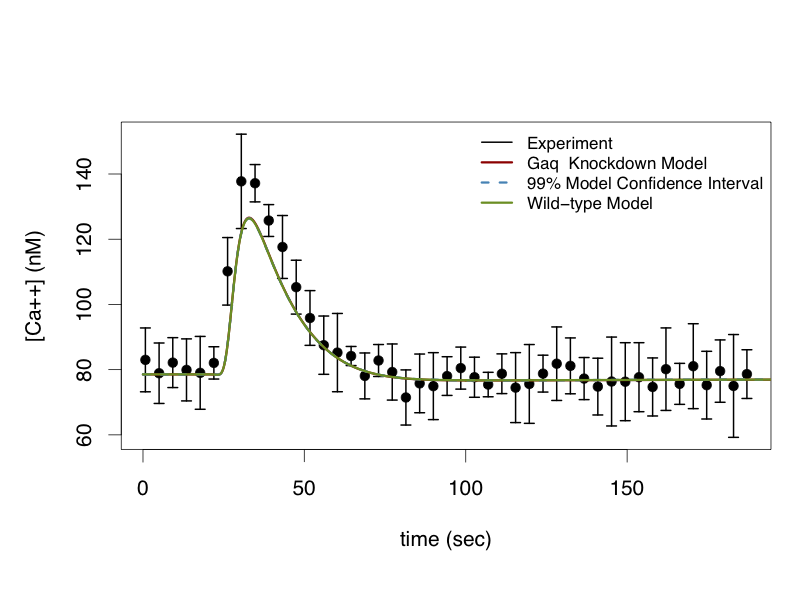 | 25μM  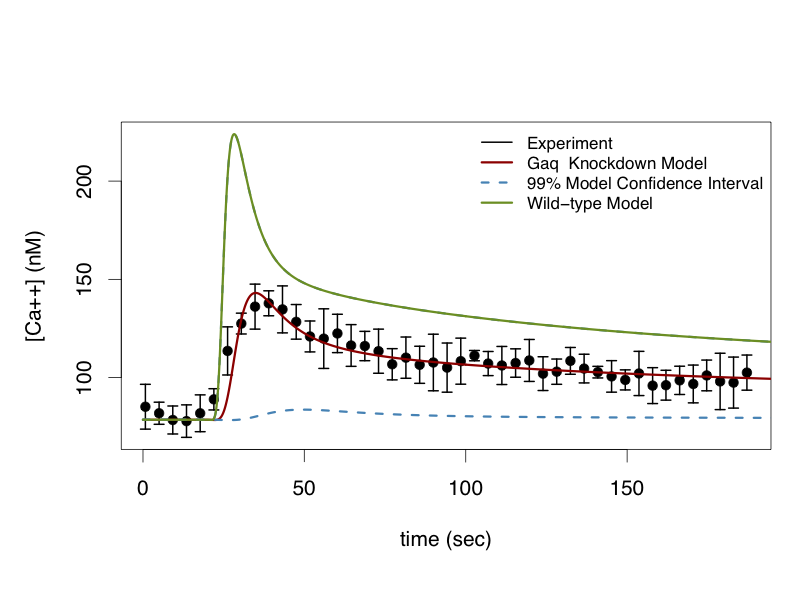 |
| PLCβ3 | 100nM  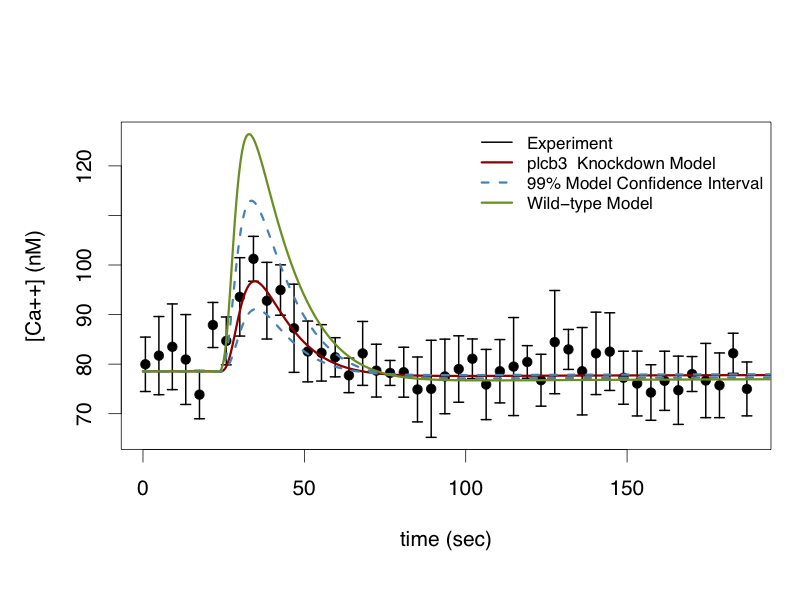 | 25μM  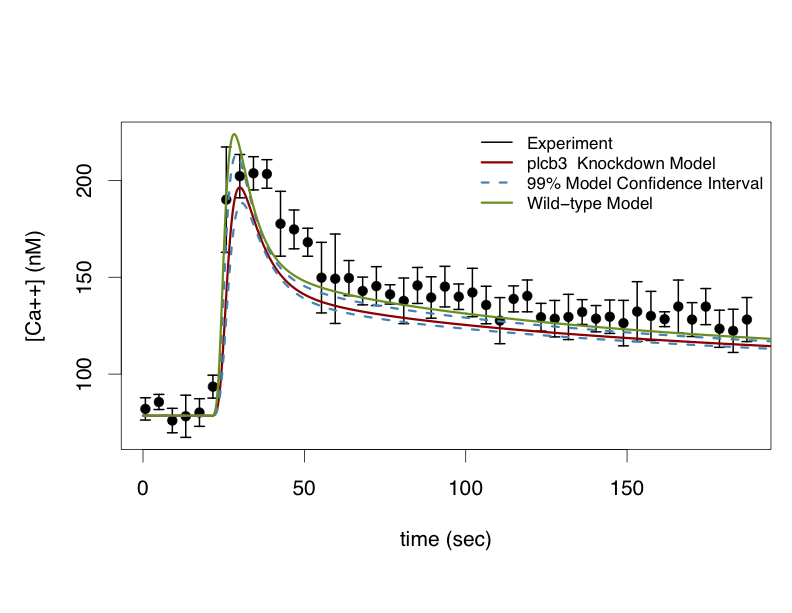 |
| PLCβ4 | 30nM  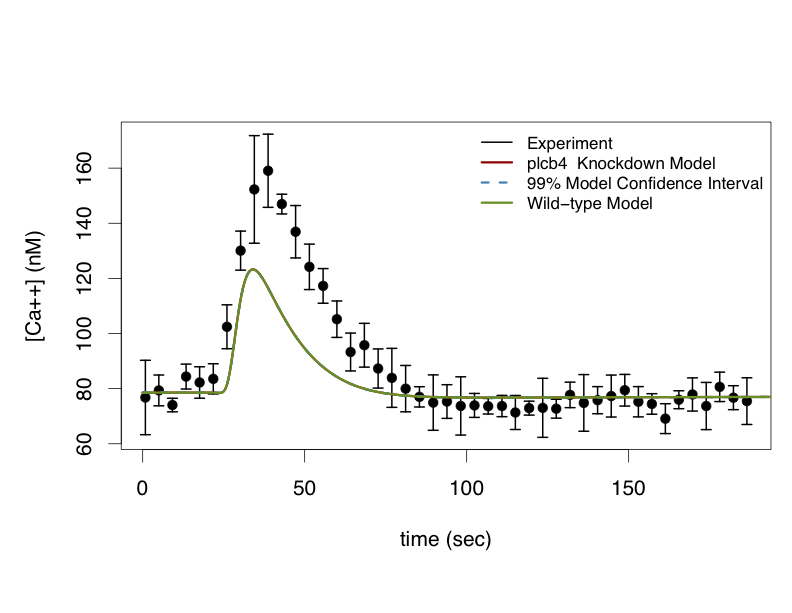 | 25μM  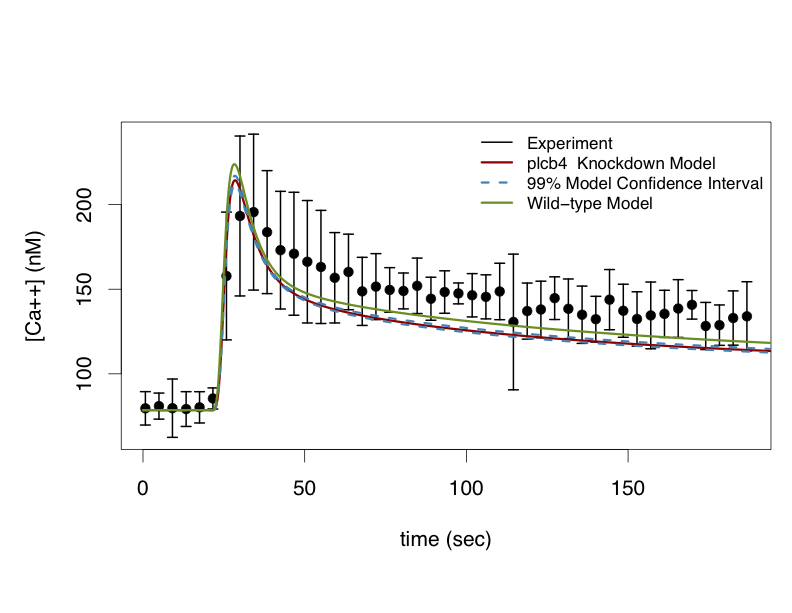 |
