# Supplementary material for: A Dual Receptor Crosstalk Model of G-Protein-Coupled Signal Transduction
Source: PLoS Comput Biol. 2008 Sep 26;4(9):e1000185. doi: 10.1371/journal.pcbi.1000185 (PMC2528964; doi:10.1371/journal.pcbi.1000185)

Figure S5: Input Model Fit

This figure shows the input model (described in Materials and Methods) fit to the FITC measurements. The ligand concentration that the cell sees does not transit instantaneously from 0 to the final concentration. The ligand concentration is expected to take an amount of time that is significant on the scale of the measurements made for this study to reach the final concentration.


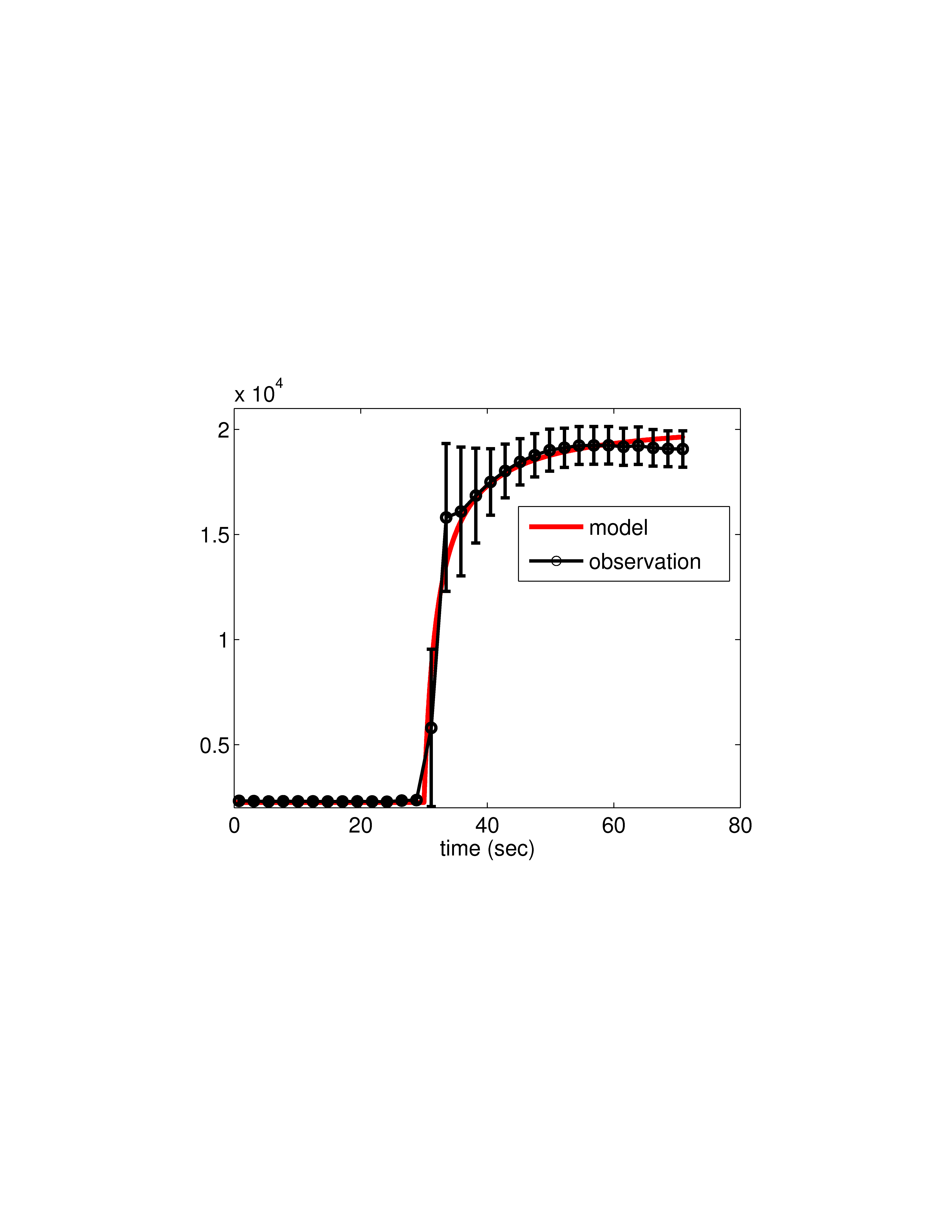

Supplement: Figure S5 — Input model fit. This figure shows the input model (described in Materials and Methods) fit to the FITC measurements. The ligand concentration that the cell sees does not transit instantaneously from 0 to the final concentration. The ligand concentration is expected to take an amount of time that is significant on the scale of the measurements made for this study to reach the final concentration. (0.12 MB DOC) [file pcbi.1000185.s006.doc]
