# Supplementary figures and images for: A Dual Receptor Crosstalk Model of G-Protein-Coupled Signal Transduction
Source: PLoS Comput Biol. 2008 Sep 26;4(9):e1000185. doi: 10.1371/journal.pcbi.1000185 (PMC2528964; doi:10.1371/journal.pcbi.1000185)

Figure S6: Large Pathway Diagram


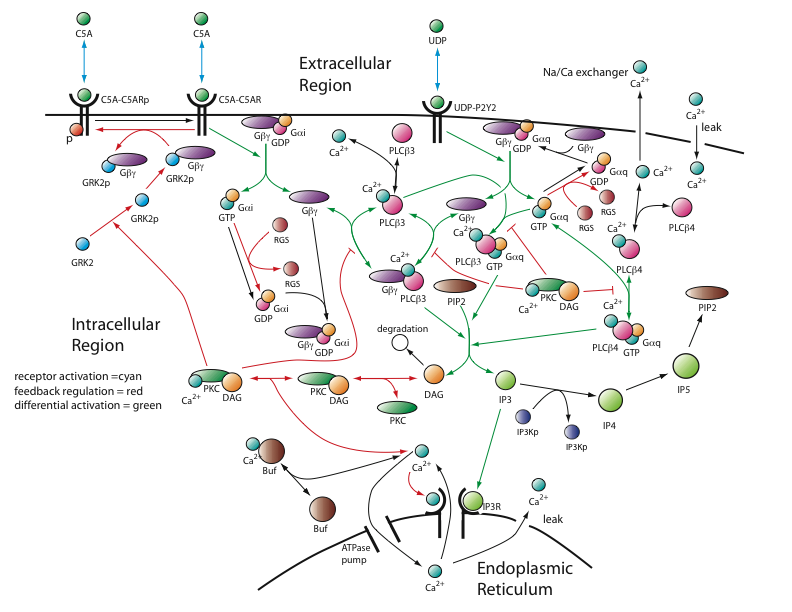

Supplement: Figure S6 — Large pathway diagram. (0.18 MB DOC) [file pcbi.1000185.s007.doc]
