# Supplementary material for: A Dual Receptor Crosstalk Model of G-Protein-Coupled Signal Transduction
Source: PLoS Comput Biol. 2008 Sep 26;4(9):e1000185. doi: 10.1371/journal.pcbi.1000185 (PMC2528964; doi:10.1371/journal.pcbi.1000185)

Figure S7: System of Differential Equations

This figure shows the complete set of differential equations used to simulate the model. These equations are also available in the source c code for the model supplied. This system of equations with the initial conditions and nominal parameter values reported in Tables S1 and S2 respectively completely define the model and allow for the reproduction of the simulations used in this paper on any platform.


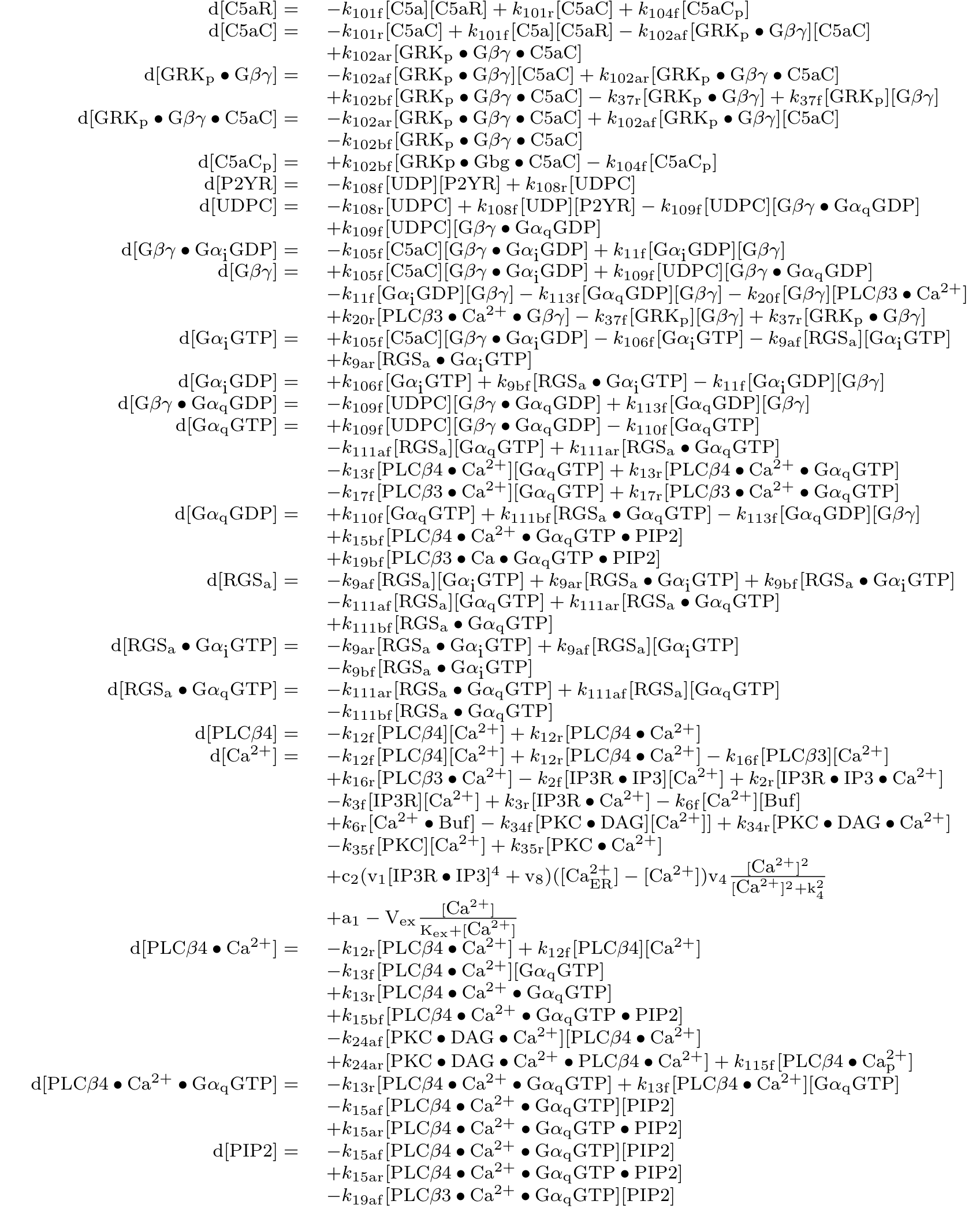


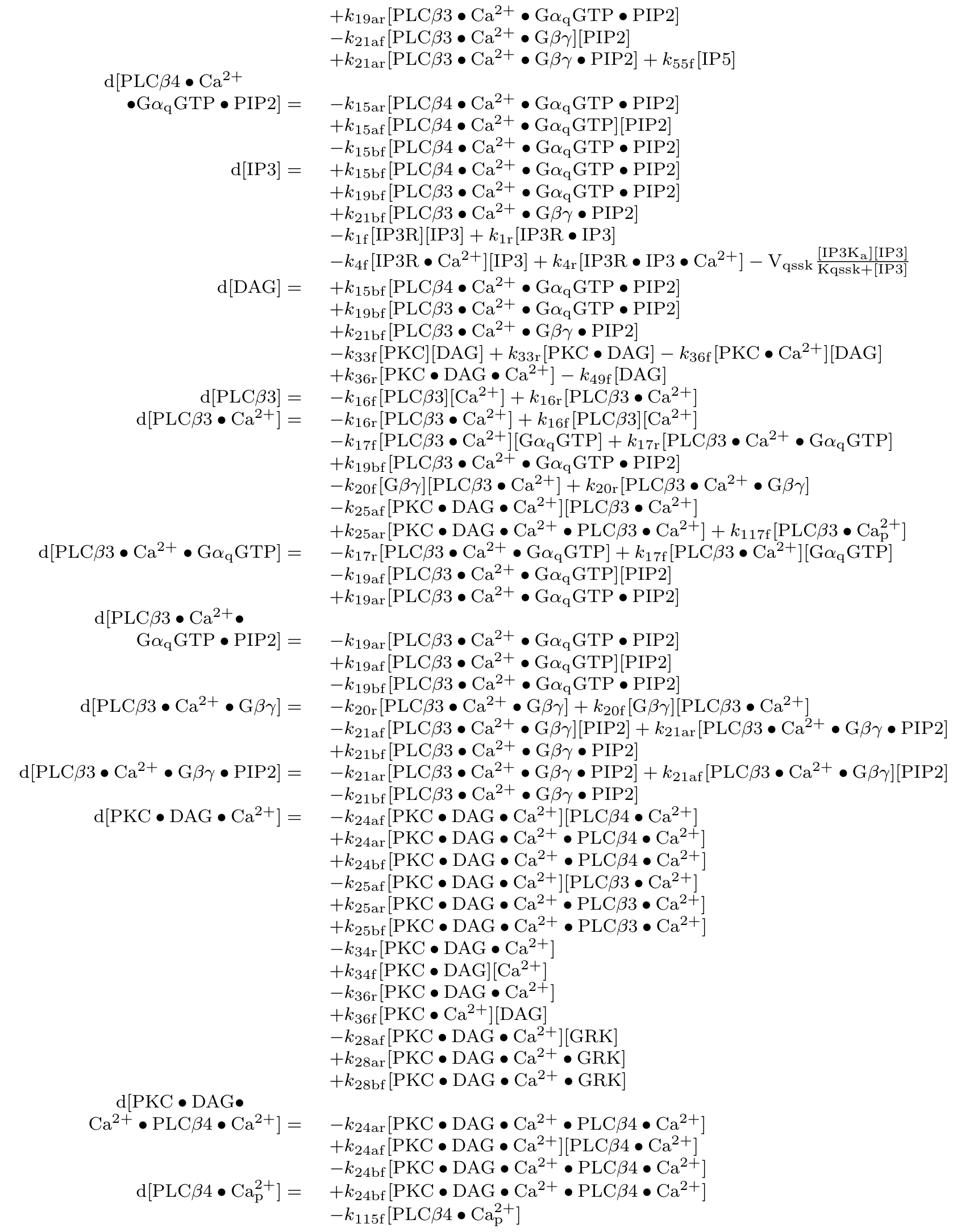


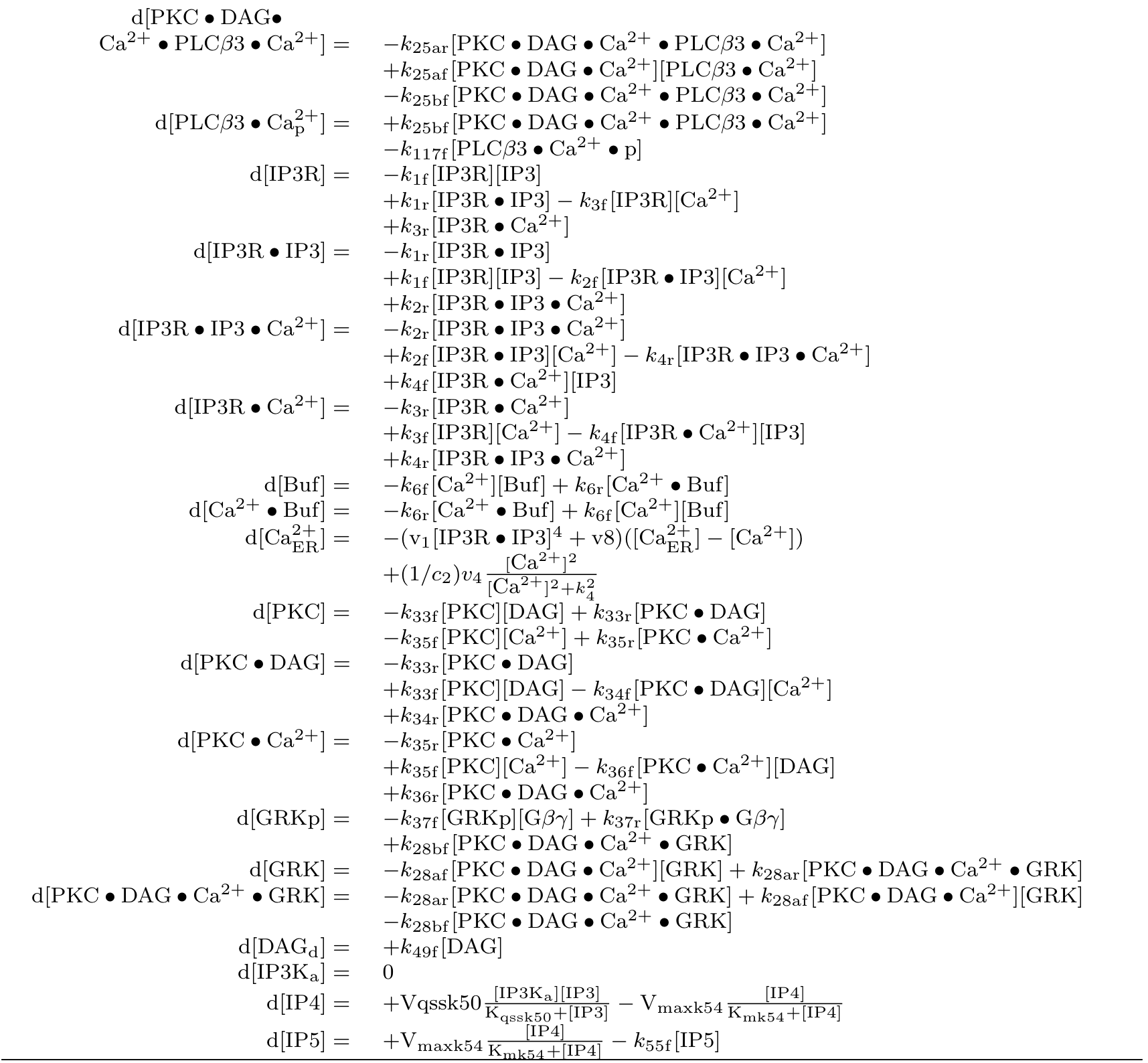

Supplement: Figure S7 — System of differential equations. This figure shows the complete set of differential equations used to simulate the model. These equations are also available in the source c code for the model supplied. This system of equations with the initial conditions and nominal parameter values reported in Table S1 and Table S2, respectively, completely define the model and allow for the reproduction of the simulations used in this paper on any platform. (1.62 MB DOC) [file pcbi.1000185.s008.doc]
