# Supplementary material for: A Dual Receptor Crosstalk Model of G-Protein-Coupled Signal Transduction
Source: PLoS Comput Biol. 2008 Sep 26;4(9):e1000185. doi: 10.1371/journal.pcbi.1000185 (PMC2528964; doi:10.1371/journal.pcbi.1000185)
Supplement: Figure S8 — Hill function self-synergy. Consider a Hill function, . is a dimensionless critical concentration y*, below which self-synergy will occur. Based on the analysis, we conclude that: (i) n must be greater than 1 for self-synergy to occur, (ii) self synergy never occurs if the concentration x exceeds equilibrium constant K (y>1), and (iii) for n>2, there is a large range of concentration for self-synergy. In the G protein model, x, is the concentration of IP3-IP3R, H(x) is the rate of change in cytosolic calcium concentration and n = 4. We have tested the validity of this self synergy hypothesis by stimulating the cells with both 20 nM UDP and 40 nM UDP (data not shown). Though at such low ligand concentrations, the measurement variability is high, we observed that the synergy ratio, on average was 1.17 compared to a value of 1.25 predicted by the model. (0.02 MB DOC) [file pcbi.1000185.s009.doc]

Figure S8: Hill function self-synergy. Consider a Hill function, $H(x)=\frac{x^{n}}{x^{n}+K^{n}}$. $y*=\frac{x*}{K}$is a dimensionless critical concentration $y*$ , below which self-synergy will occur. Based on the analysis, we conclude that: (i) *n* must be greater than 1 for self-synergy to occur, (ii) self synergy never occurs if the concentration *x* exceeds equilibrium constant *K* (y > 1), and (iii) for *n > 2*, there is a large range of concentration for self-synergy. In the G protein model, x, is the concentration of IP3-IP3R, H(x) is the rate of change in cytosolic calcium concentration and n=4.

We have tested the validity of this self synergy hypothesis by stimulating the cells with both 20nM UDP and 40nM UDP (data not shown). Though at such low ligand concentrations, the measurement variability is high, we observed that the synergy ratio, on average was 1.17 compared to a value of 1.25 predicted by the model.
