# Supplementary material for: A Dual Receptor Crosstalk Model of G-Protein-Coupled Signal Transduction
Source: PLoS Comput Biol. 2008 Sep 26;4(9):e1000185. doi: 10.1371/journal.pcbi.1000185 (PMC2528964; doi:10.1371/journal.pcbi.1000185)

Figure S9: Parameter Sensitivity Analysis. The parameter of interest is varied by 10% while all other parameters are kept constant. The parameters are grouped according to their functionalities. The sensitivity coefficient is the ratio of the relative change in the peak height to the relative change in the parameter value. The four most sensitive parameters (sensitivity coefficient > 2) in the Ca_cyt_ category are Vqssk50 (IP3+IP3K_a -> IP4+IP3K_a (Vmax)), Kqssk50 (IP3+IP3K_a -> IP4+IP3K_a (Km)), a1 (Ca leak into the cell from outside), and Kex (Na/Ca exchange activation const). The top 3 most sensitive parameters in the PLCb3 category are: k21bf* (PLCb3_Ca_Gbg_PIP2 -> PLCb3_Ca_Gbg+IP3+DAG), k20f (Gbg+PLCb3_Ca -> PLCb3_Ca_Gbg), k21af* (PLCb3_Ca_Gbg+PIP2 -> PLCb3_Ca_Gbg_PIP2). A star next to the parameter name indicates it was estimated.


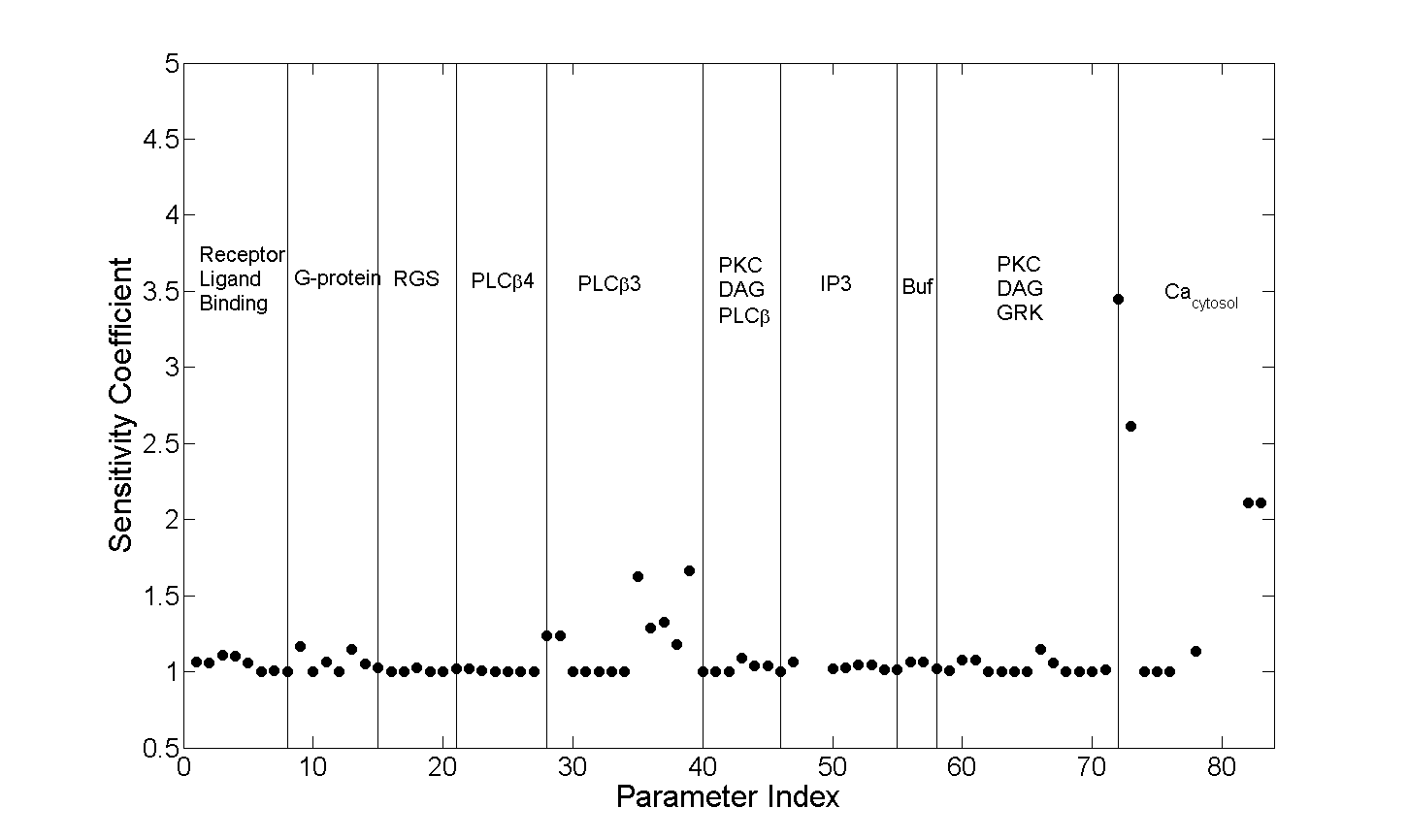

Supplement: Figure S9 — Parameter sensitivity analysis. The parameter of interest is varied by 10% while all other parameters are kept constant. The parameters are grouped according to their functionalities. The sensitivity coefficient is the ratio of the relative change in the peak height to the relative change in the parameter value. The four most sensitive parameters (sensitivity coefficient >2) in the Cacyt category are Vqssk50 (IP3+IP3K_a->IP4+IP3K_a (Vmax)), Kqssk50 (IP3+IP3K_a->IP4+IP3K_a (Km)), a1 (Ca leak into the cell from outside), and Kex (Na/Ca exchange activation const). The top 3 most sensitive parameters in the PLCb3 category are: k21bf* (PLCb3_Ca_Gbg_PIP2->PLCb3_Ca_Gbg+IP3+DAG), k20f (Gbg+PLCb3_Ca->PLCb3_Ca_Gbg), k21af* (PLCb3_Ca_Gbg+PIP2->PLCb3_Ca_Gbg_PIP2). A star next to the parameter name indicates it was estimated. (0.04 MB DOC) [file pcbi.1000185.s010.doc]
