# Supplementary material for: A Dual Receptor Crosstalk Model of G-Protein-Coupled Signal Transduction
Source: PLoS Comput Biol. 2008 Sep 26;4(9):e1000185. doi: 10.1371/journal.pcbi.1000185 (PMC2528964; doi:10.1371/journal.pcbi.1000185)
Supplement: Protocol S1 — FITC protocol. (0.03 MB DOC) [file pcbi.1000185.s011.doc]

Protocol 1: FITC Protocol

1. FITC soln at 400nM used for robotic addition to wells.  A 4-fold in-well
   dilution yielded 100nM final in a final 100ul well volume.
2. Wells contained 75 ul water.
3. Instrument settings:
           Flex mode = serial reads at FITC wavelengths (495ex/ 525em, 510
   cutoff)
           Robotic additions at 20 seconds
           Additions defined by time / volume / ejection height / ejection
   speed plus
                   trituration # cycles / volume / height (speed is defined
   by above)
4. Settings for first vs second data set were:
           25ul volume / 75ul height / speed 1
           25ul volume / 50ul height / speed 4 plus 1 trituration @ 25ul
   volume, 50ul height
