# Supplementary material for: A Dual Receptor Crosstalk Model of G-Protein-Coupled Signal Transduction
Source: PLoS Comput Biol. 2008 Sep 26;4(9):e1000185. doi: 10.1371/journal.pcbi.1000185 (PMC2528964; doi:10.1371/journal.pcbi.1000185)
Supplement: Table S1 — Model initial conditions. This table shows the initial conditions used for the model. The model was run for sufficient time for the species states in the model to reach equilibrium before ligand stimulation was added. The number of molecules was calculated using a cell volume of 1 pL. (0.05 MB DOC) [file pcbi.1000185.s012.doc]

Table S1: Model Initial Conditions

This table shows the initial conditions used for the model. The model was run for sufficient time for the species states in the model to reach equilibrium before ligand stimulation was added. The number of molecules was calculated using a cell volume of 1pL.

| **Name** | **Initial Value (μM)** | **Molecules** | **Description** |
| --- | --- | --- | --- |
| c5aR | 5.00E-02 | 30100 | C5a receptor concentration |
| p2yr | 1.00E-01 | 60200 | P2Y receptor concentration |
| Gβγ | 7.14E+00 | 4299990 | Gβγ concentration |
| Gαi_GDP | 6.64E+00 | 3999989 | Gαi concentration |
| Gαq_GDP | 4.98E-01 | 300001 | Gαq concentration |
| PLCβ3 | 1.16E-01 | 70001 | PLCβ3 concentration |
| PLCβ4 | 6.64E-02 | 40000 | PLCβ4 concentration |
| PIP2 | 5.00E-01 | 301000 |  |
| IP3 | 1.80E-03 | 1084 | Free IP3 concentration |
| DAG | 1.00E-03 | 602 | Free DAG concentration |
| IP3R | 2.08E-02 | 12492 | IP3 receptor concentration |
| IP3R-IP3 | 1.75E-03 | 1054 |  |
| IP3R-IP3-Ca | 2.30E-03 | 1385 |  |
| IP3R-Ca | 2.00E-04 | 120 |  |
| Ca | 7.86E-02 | 47317 | Cytosolic Calcium concentration |
| CaER | 1.04E+01 | 6231302 | IP3 sensitive stored calcium concentration |
| PKC | 2.49E-02 | 15000 |  |
| GRK | 2.31E-02 | 13880 | GRK concentration |
| RGS_a | 2.31E-02 | 13880 | Regulator of G protein Signaling |
| Buf | 4.50E-01 | 270599 |  |
| CaBuf | 5.05E-02 | 30401 |  |
| IP3K_a | 1.66E-03 | 1000 |  |
| IP4 | 1.00E-01 | 60200 |  |
| IP5 | 1.00E-01 | 60200 |  |
