# Supplementary material for: A Dual Receptor Crosstalk Model of G-Protein-Coupled Signal Transduction
Source: PLoS Comput Biol. 2008 Sep 26;4(9):e1000185. doi: 10.1371/journal.pcbi.1000185 (PMC2528964; doi:10.1371/journal.pcbi.1000185)
Supplement: Table S2 — Model parameters. This table shows the nominal parameters used for the model. Parameter distributions that were estimated are shown as shaded rows and with a star next to the parameter name in the table. The prior distribution for each parameter is as described in the Materials and Methods section with mean value specified by the column labeled “prior”. (0.14 MB DOC) [file pcbi.1000185.s013.doc]

Table S2: Model Parameters

This table shows the nominal parameters used for the model. Parameter distributions that were estimated are shown as shaded rows and with a star next to the parameter name in the table. The prior distribution for each parameter is as described in the Materials and Methods section with mean value specified by the column labeled "prior".

| **Constant** | **Prior** | **Nominal** | **Unit** | **Description** |
| --- | --- | --- | --- | --- |
| k108f* | 1.628 | 13.20 | μM-1 s-1 | UDP+p2yr -> UDPC |
| k108r* | 0.165 | 3.61 | s-1 | UDP+p2yr <- UDPC |
| k101f* | 12.143 | 92.41 | μM-1 s-1 | c5a+c5aR -> c5aC |
| k101r* | 0.0378 | 0.376 | s-1 | c5a+c5aR <- c5aC |
| k102af | 591.54 | 591.54 | μM-1s-1 | GRKp_Gbg+c5aC -> GRKp_Gbg_c5aC |
| k102ar | 12.367 | 12.36 | s-1 | GRKp_Gbg+c5aC <- GRKp_Gbg_c5aC |
| k102bf* | 123.31 | 199.31 | s-1 | GRKp_Gbg_c5aC -> GRKp_Gbg+c5aCp |
| k104f | 0.0001 | 0.0001 | s-1 | c5aCp -> c5aR+c5a |
| k105f* | 0.0945 | 0.012 | μM-1 s-1 | c5aC+Gbg_Gai_GDP -> c5aC+Gbg+Gai_GTP |
| k106f | 0.0222 | 0.0222 | s-1 | Gai_GTP -> Gai_GDP |
| k109f* | 0.2686 | 0.137 | μM-1 s-1 | UDPC+Gbg_Gaq_GDP -> UDPC+Gbg+Gaq_GTP |
| k110f | 0.0222 | 0.0222 | s-1 | Gaq_GTP -> Gaq_GDP |
| k11f | 7000 | 7000 | μM-1 s-1 | Gai_GDP+Gbg -> Gbg_Gai_GDP |
| k113f | 7000 | 7000 | μM-1 s-1 | Gaq_GDP+Gbg -> Gbg_Gaq_GDP |
| k9af | 100 | 100 | μM-1 s-1 | RGS_a+Gai_GTP -> RGS_a_Gai_GTP |
| k9ar | 0.1 | 0.1 | s-1 | RGS_a+Gai_GTP <- RGS_a_Gai_GTP |
| k9bf | 100 | 100 | s-1 | RGS_a_Gai_GTP -> RGS_a+Gai_GDP |
| k111af | 100 | 100 | μM-1 s-1 | RGS_a+Gaq_GTP -> RGS_a_Gaq_GTP |
| k111ar | 0.1 | 0.1 | s-1 | RGS_a+Gaq_GTP <- RGS_a_Gaq_GTP |
| k111bf | 100 | 100 | s-1 | RGS_a_Gaq_GTP -> RGS_a+Gaq_GDP |
| k12f | 20 | 20 | μM-1 s-1 | PLCb4+Ca -> PLCb4_Ca |
| k12r | 8 | 8 | s-1 | PLCb4+Ca <- PLCb4_Ca |
| k13f | 62.55 | 62.55 | μM-1 s-1 | PLCb4_Ca+Gaq_GTP -> PLCb4_Ca_Gaq_GTP |
| k13r | 10.632 | 10.63 | s-1 | PLCb4_Ca+Gaq_GTP <- PLCb4_Ca_Gaq_GTP |
| k15af* | 100 | 1238.78 | μM-1 s-1 | PLCb4_Ca_Gaq_GTP+PIP2 -> PLCb4_Ca_Gaq_GTP_PIP2 |
| k15ar | 1 | 1 | s-1 | PLCb4_Ca_Gaq_GTP+PIP2 <- PLCb4_Ca_Gaq_GTP_PIP2 |
| k15bf* | 3 | 22.85 | s-1 | PLCb4_Ca_Gaq_GTP_PIP2 -> PLCb4_Ca+Gaq_GDP+IP3+DAG |
| k16f | 20 | 20 | μM-1 s-1 | PLCb3+Ca -> PLCb3_Ca |
| k16r | 8 | 8 | s-1 | PLCb3+Ca <- PLCb3_Ca |
| k17f | 50 | 50 | μM-1 s-1 | PLCb3_Ca+Gaq_GTP -> PLCb3_Ca_Gaq_GTP |
| k17r | 0.1 | 0.1 | s-1 | PLCb3_Ca+Gaq_GTP <- PLCb3_Ca_Gaq_GTP |
| k19af* | 100 | 70.87 | μM-1 s-1 | PLCb3_Ca_Gaq_GTP+PIP2 -> PLCb3_Ca_Gaq_GTP_PIP2 |
| k19ar | 1 | 1 | s-1 | PLCb3_Ca_Gaq_GTP+PIP2 <- PLCb3_Ca_Gaq_GTP_PIP2 |
| k19bf* | 3 | 27.89 | s-1 | PLCb3_Ca_Gaq_GTP_PIP2 -> PLCb3_Ca+Gaq_GDP+IP3+DAG |
| k20f | 8.346 | 8.346 | μM-1 s-1 | Gbg+PLCb3_Ca -> PLCb3_Ca_Gbg |
| k20r | 0.388 | 0.388 | s-1 | Gbg+PLCb3_Ca <- PLCb3_Ca_Gbg |
| k21af* | 80 | 165.83 | μM-1 s-1 | PLCb3_Ca_Gbg+PIP2 -> PLCb3_Ca_Gbg_PIP2 |
| k21ar | 8 | 8 | s-1 | PLCb3_Ca_Gbg+PIP2 <- PLCb3_Ca_Gbg_PIP2 |
| k21bf* | 1 | 5.41 | s-1 | PLCb3_Ca_Gbg_PIP2 -> PLCb3_Ca_Gbg+IP3+DAG |
| k24af | 10 | 5.89 | μM-1 s-1 | PKC_DAG_Ca+PLCb4_Ca -> PKC_DAG_Ca_PLCb4_Ca |
| k24ar | 11 | 11 | s-1 | PKC_DAG_Ca+PLCb4_Ca <- PKC_DAG_Ca_PLCb4_Ca |
| k24bf | 1 | 0.93 | s-1 | PKC_DAG_Ca_PLCb4_Ca -> PKC_DAG_Ca+PLCb4_Ca_p |
| k25af | 110 | 830.44 | μM-1 s-1 | PKC_DAG_Ca+PLCb3_Ca -> PKC_DAG_Ca_PLCb3_Ca |
| k25ar | 11 | 11 | s-1 | PKC_DAG_Ca+PLCb3_Ca <- PKC_DAG_Ca_PLCb3_Ca |
| k25bf | 1 | 11.69 | s-1 | PKC_DAG_Ca_PLCb3_Ca -> PKC_DAG_Ca+PLCb3_Ca_p |
| k115f | 0.12 | 0.12 | s-1 | PLCb4_Ca_p -> PLCb4_Ca |
| k117f | 0.12 | 0.12 | s-1 | PLCb3_Ca_p -> PLCb3_Ca |
| k1f | 177.47 | 177.47 | μM-1 s-1 | IP3R+IP3 -> IP3R_IP3 |
| k1r | 2.2 | 2.2 | s-1 | IP3R+IP3 <- IP3R_IP3 |
| k2f | 0.411 | 0.411 | μM-1 s-1 | IP3R_IP3+Ca -> IP3R_IP3_Ca |
| k2r | 0.0434 | 0.0434 | s-1 | IP3R_IP3+Ca <- IP3R_IP3_Ca |
| k3f | 0.9 | 0.9 | μM-1 s-1 | IP3R+Ca -> IP3R_Ca |
| k3r | 0.806 | 0.806 | s-1 | IP3R+Ca <- IP3R_Ca |
| k4f | 20 | 20 | μM-1 s-1 | IP3R_Ca+IP3 -> IP3R_IP3_Ca |
| k4r | 0.029 | 0.029 | s-1 | IP3R_Ca+IP3 <- IP3R_IP3_Ca (thermcycle) |
| k6f | 10 | 10 | μM-1 s-1 | Ca+Buf -> CaBuf |
| k6r | 7 | 7 | s-1 | Ca+Buf <- CaBuf |
| k33f | 100 | 100 | μM-1 s-1 | PKC+DAG -> PKC_DAG |
| k33r | 0.05 | 0.05 | s-1 | PKC+DAG <- PKC_DAG |
| k34f | 10 | 10 | μM-1 s-1 | PKC_DAG+Ca -> PKC_DAG_Ca |
| k34r | 6 | 6 | s-1 | PKC_DAG+Ca <- PKC_DAG_Ca (thermcycle) |
| k35f | 0.01 | 0.01 | μM-1 s-1 | PKC+Ca -> PKC_Ca |
| k35r | 30 | 30 | s-1 | PKC+Ca <- PKC_Ca |
| k36f | 1000 | 1000 | μM-1 s-1 | PKC_Ca+DAG -> PKC_DAG_Ca |
| k36r | 0.0001 | 0.0001 | s-1 | PKC_Ca+DAG <- PKC_DAG_Ca |
| k37f | 1 | 4.98 | μM-1 s-1 | GRKp+Gbg -> GRKp_Gbg |
| k37r | 0.05 | 0.05 | s-1 | GRKp+Gbg <- GRKp_Gbg |
| k28af | 158.49 | 77.52 | μM-1 s-1 | PKC_DAG_Ca+GRK -> PKC_DAG_Ca_GRK |
| k28ar | 10 | 10 | s-1 | PKC_DAG_Ca+GRK <- PKC_DAG_Ca_GRK |
| k28bf | 10 | 18.34 | s-1 | PKC_DAG_Ca_GRK -> PKC_DAG_Ca+GRKp |
| k49f | 0.35 | 0.35 | s-1 | DAG -> DAG_d |
| Vqssk50 | 13.9 | 13.9 | s-1 | IP3+IP3K_a -> IP4+IP3K_a (Vmax) |
| Kqssk50 | 0.055 | 0.055 | μM | IP3+IP3K_a -> IP4+IP3K_a (Km) |
| Vmaxk54 | 100 | 100 | μM s-1 | IP4 -> IP5 |
| Kmk54 | 1.4 | 1.4 | μM | IP4 -> IP5 |
| k55f | 0.008 | 0.008 | s-1 | IP5 -> PIP2 |
| c2 | 0.185 | 0.185 | none | ratio of ER volume/cell: de young |
| v1 | 1E8 | 1E8 | s-1 | Ca channel flux constant |
| v8 | 0.15 | 0.15 | s-1 | leak flux constant |
| v4 | 20 | 20 | μM s-1 | maximum Ca uptake rate (SERCA) |
| k4 | 0.65 | 0.65 | μM | activation constant of SERCA pump |
| a1 | 0.0055 | 0.0055 | μM s-1 | Ca leak into the cell from outside |
| Kex | 0.25 | 0.25 | μM | Na/Ca exchange activation const |
| Vex | 0.023 | 0.023 | μM s-1 | maximum Ca exchange rate |
