# Supplementary material for: A Dual Receptor Crosstalk Model of G-Protein-Coupled Signal Transduction
Source: PLoS Comput Biol. 2008 Sep 26;4(9):e1000185. doi: 10.1371/journal.pcbi.1000185 (PMC2528964; doi:10.1371/journal.pcbi.1000185)
Supplement: Table S3 — Parameter posterior uncertainty and references. This table shows the HPD intervals as computed by the R CODA library function “hpdinterval”. HPD intervals for each of the three MCMC chains were calculated and the union of those intervals is reported for each parameter in this table. The prior value reported in Table S2 was set using information from references listed in the appropriate column. The references used to form the basis of the parameter estimates are shown in the last column. (0.37 MB DOC) [file pcbi.1000185.s014.doc]

Table S3: Parameter Posterior Uncertainty and References

This table shows the HPD intervals as computed by the R CODA library function "hpdinterval". HPD intervals for each of the three MCMC chains were calculated and the union of those intervals is reported for each parameter in this table. The prior value reported in Table S2 was set using information from references listed in the appropriate column. The references used to form the basis of the parameter estimates are shown in the last column. A detailed discussion of the choice of rate constant estimates is shown in (3).

|  |  | Parameter HPD Interval | | | |  |
| --- | --- | --- | --- | --- | --- | --- |
|  |  | mean | lower 95% | upper 95% | model | Reference |
| k108f* | UDP+p2yr -> UDPC | 12.85 | 6.64 | 18.47 | 13.20 |  |
| k108r* | UDP+p2yr <- UDPC | 3.54 | 1.88 | 5.01 | 3.62 |  |
| k101f* | c5a+c5aR -> c5aC | 134.61 | 32.34 | 197.77 | 92.41 |  |
| k101r* | c5a+c5aR <- c5aC | 0.67 | 0.04 | 1.00 | 0.38 |  |
| k102af | GRKp_Gbg+c5aC -> GRKp_Gbg_c5aC | 591.54 | 591.54 | 591.54 | 591.54 |  |
| k102ar | GRKp_Gbg+c5aC <- GRKp_Gbg_c5aC | 12.37 | 12.37 | 12.37 | 12.37 |  |
| k102bf* | GRKp_Gbg_c5aC <- GRKp_Gbg+c5aCp | 378.66 | 118.54 | 660.84 | 199.31 |  |
| k104f | c5aCp -> c5aR+c5a | 1.00E-04 | 1.00E-04 | 1.00E-04 | 1.00E-04 |  |
| k105f* | c5aC+Gbg_Gai_GDP -> c5aC+Gbg+Gai_GTP | 1.30E-02 | 1.09E-02 | 1.79E-02 | 1.29E-02 |  |
| k106f | Gai_GTP -> Gai_GDP | 0.02 | 0.02 | 0.02 | 0.02 |  |
| k109f* | UDPC+Gbg_Gaq_GDP -> UDPC+Gbg+Gaq_GTP | 1.36E-01 | 1.26E-01 | 1.51E-01 | 1.37E-01 |  |
| k110f | Gaq_GTP -> Gaq_GDP | 0.02 | 0.02 | 0.02 | 0.02 |  |
| k11f | Gai_GDP+Gbg -> Gbg_Gai_GDP | 7000.00 | 7000.00 | 7000.00 | 7000.00 |  |
| k113f | Gaq_GDP+Gbg -> Gbg_Gaq_GDP | 7000.00 | 7000.00 | 7000.00 | 7000.00 |  |
| k9af | RGS_a+Gai_GTP -> RGS_a_Gai_GTP | 100.00 | 100.00 | 100.00 | 100.00 |  |
| k9ar | RGS_a+Gai_GTP <- RGS_a_Gai_GTP | 0.10 | 0.10 | 0.10 | 0.10 |  |
| k9bf | RGS_a_Gai_GTP -> RGS_a+Gai_GDP | 100.00 | 100.00 | 100.00 | 100.00 |  |
| k111af | RGS_a+Gaq_GTP -> RGS_a_Gaq_GTP | 100.00 | 100.00 | 100.00 | 100.00 |  |
| k111ar | RGS_a+Gaq_GTP <- RGS_a_Gaq_GTP | 0.10 | 0.10 | 0.10 | 0.10 |  |
| k111bf | RGS_a_Gaq_GTP -> RGS_a+Gaq_GDP | 100.00 | 100.00 | 100.00 | 100.00 |  |
| k12f | PLCb4+Ca -> PLCb4_Ca | 20.00 | 20.00 | 20.00 | 20.00 | (4, 5) |
| k12r | PLCb4+Ca <- PLCb4_Ca | 8.00 | 8.00 | 8.00 | 8.00 | (4, 5) |
| k13f | PLCb4_Ca+Gaq_GTP -> PLCb4_Ca_Gaq_GTP | 62.55 | 62.55 | 62.55 | 62.55 | (6, 7) |
| k13r | PLCb4_Ca+Gaq_GTP <- PLCb4_Ca_Gaq_GTP | 10.63 | 10.63 | 10.63 | 10.63 | (6, 7) |
| k15af* | PLCb4_Ca_Gaq_GTP+PIP2 -> PLCb4_Ca+Gaq_GTP_PIP2 | 1497.23 | 764.53 | 3041.57 | 1238.79 | (6, 7) |
| k15ar | PLCb4_Ca_Gaq_GTP+PIP2 <- PLCb4_Ca+Gaq_GTP_PIP2 | 1.00 | 1.00 | 1.00 | 1.00 | (6, 7) |
| k15bf* | PLCb4_Ca+Gaq_GTP_PIP2 -> PLCb4_Ca+Gaq_GDP+IP3+DAG | 24.77 | 12.11 | 69.52 | 22.85 | (6, 7) |
| k16f | PLCb3+Ca -> PLCb3_Ca | 20.00 | 20.00 | 20.00 | 20.00 | (4, 5, 8, 9) |
| k16r | PLCb3+Ca <- PLCb3_Ca | 8.00 | 8.00 | 8.00 | 8.00 | (4, 5, 8, 9) |
| k17f | PLCb3_Ca+Gaq_GTP -> PLCb3_Ca_Gaq_GTP | 50.00 | 50.00 | 50.00 | 50.00 | (10, 11) |
| k17r | PLCb3_Ca+Gaq_GTP <- PLCb3_Ca_Gaq_GTP | 0.10 | 0.10 | 0.10 | 0.10 | (10, 11) |
| k19af* | PLCb3_Ca_Gaq_GTP+PIP2 -> PLCb3_Ca_Gaq_GTP_PIP2 | 176.85 | 30.58 | 302.61 | 70.88 | (10) |
| k19ar | PLCb3_Ca_Gaq_GTP+PIP2 <- PLCb3_Ca_Gaq_GTP_PIP2 | 1.00 | 1.00 | 1.00 | 1.00 | (10) |
| k19bf* | PLCb3_Ca_Gaq_GTP_PIP2 -> PLCb3_Ca+Gaq_GDP+IP3+DAG | 32.19 | 12.24 | 56.67 | 27.90 | (10) |
| k20f | Gbg+PLCb3_Ca -> PLCb3_Ca_Gbg | 8.35 | 8.35 | 8.35 | 8.35 | (10, 12-15) |
| k20r | Gbg+PLCb3_Ca <- PLCb3_Ca_Gbg | 0.39 | 0.39 | 0.39 | 0.39 | (10, 12-15) |
| k21af* | PLCb3_Ca_Gbg+PIP2 -> PLCb3_Ca_Gbg_PIP2 | 162.54 | 149.74 | 179.55 | 165.83 | (10, 12-15) |
| k21ar | PLCb3_Ca_Gbg+PIP2 <- PLCb3_Ca_Gbg_PIP2 | 8.00 | 8.00 | 8.00 | 8.00 | (10, 12-15) |
| k21bf* | PLCb3_Ca_Gbg_PIP2 -> PLCb3_Ca_Gbg+IP3+DAG | 5.49 | 5.09 | 5.90 | 5.42 | (10, 12-15) |
| k24af* | PKC_DAG_Ca+PLCb4_Ca -> PKC_DAG_Ca_PLCb4_Ca | 5.92 | 2.31 | 12.55 | 5.90 |  |
| k24ar | PKC_DAG_Ca+PLCb4_Ca <- PKC_DAG_Ca_PLCb4_Ca | 11.00 | 11.00 | 11.00 | 11.00 |  |
| k24bf* | PKC_DAG_Ca_PLCb4_Ca -> PKC_DAG_Ca+PLCb4_Ca_p | 1.00 | 0.22 | 1.84 | 0.93 |  |
| k25af* | PKC_DAG_Ca+PLCb3_Ca -> PKC_DAG_Ca_PLCb3_Ca | 716.37 | 513.98 | 856.07 | 830.44 |  |
| k25ar | PKC_DAG_Ca+PLCb3_Ca <- PKC_DAG_Ca_PLCb3_Ca | 11.00 | 11.00 | 11.00 | 11.00 |  |
| k25bf* | PKC_DAG_Ca_PLCb3_Ca -> PKC_DAG_Ca+PLCb3_Ca_p | 15.95 | 9.04 | 32.19 | 11.70 |  |
| k115f | PLCb4_Ca_p -> PLCb4_Ca | 0.12 | 0.12 | 0.12 | 0.12 |  |
| k117f | PLCb3_Ca_p -> PLCb3_Ca | 0.12 | 0.12 | 0.12 | 0.12 |  |
| k1f | IP3R+IP3 -> IP3R_IP3 | 177.47 | 177.47 | 177.47 | 177.47 | (16, 17) |
| k1r | IP3R+IP3 <- IP3R_IP3 | 2.20 | 2.20 | 2.20 | 2.20 | (16, 17) |
| k2f | IP3R_IP3+Ca -> IP3R_IP3_Ca | 0.41 | 0.41 | 0.41 | 0.41 | (16, 17) |
| k2r | IP3R_IP3+Ca <- IP3R_IP3_Ca | 0.04 | 0.04 | 0.04 | 0.04 | (16, 17) |
| k3f | IP3R+Ca -> IP3R_Ca | 0.90 | 0.90 | 0.90 | 0.90 | (16, 17) |
| k3r | IP3R+Ca <- IP3R_Ca | 0.81 | 0.81 | 0.81 | 0.81 | (16, 17) |
| k4f | IP3R_Ca+IP3 -> IP3R_IP3_Ca | 20.00 | 20.00 | 20.00 | 20.00 | (16, 17) |
| k4r | IP3R_Ca+IP3 <- IP3R_IP3_Ca (thermcycle) | 0.03 | 0.03 | 0.03 | 0.03 | (16, 17) |
| k6f | Ca+Buf -> CaBuf | 10.00 | 10.00 | 10.00 | 10.00 |  |
| k6r | Ca+Buf <- CaBuf | 7.00 | 7.00 | 7.00 | 7.00 |  |
| k33f | PKC+DAG -> PKC_DAG | 100.00 | 100.00 | 100.00 | 100.00 | (18, 19) |
| k33r | PKC+DAG <- PKC_DAG | 0.05 | 0.05 | 0.05 | 0.05 | (18, 19) |
| k34f | PKC_DAG+Ca -> PKC_DAG_Ca | 10.00 | 10.00 | 10.00 | 10.00 | (18, 19) |
| k34r | PKC_DAG+Ca <- PKC_DAG_Ca (thermcycle) | 6.00 | 6.00 | 6.00 | 6.00 | (18, 19) |
| k35f | PKC+Ca -> PKC_Ca | 0.01 | 0.01 | 0.01 | 0.01 | (18, 19) |
| k35r | PKC+Ca <- PKC_Ca | 30.00 | 30.00 | 30.00 | 30.00 | (18, 19) |
| k36f | PKC_Ca+DAG -> PKC_DAG_Ca | 1000.00 | 1000.00 | 1000.00 | 1000.00 | (18, 19) |
| k36r | PKC_Ca+DAG <- PKC_DAG_Ca | 0.00 | 0.00 | 0.00 | 1.00E-04 | (18, 19) |
| k37f* | GRKp+Gbg -> GRKp_Gbg | 4.76 | 3.97 | 6.94 | 4.98 | (20, 21) |
| k37r | GRKp+Gbg <- GRKp_Gbg | 0.05 | 0.05 | 0.05 | 0.05 | (20, 21) |
| k28af* | PKC_DAG_Ca+GRK -> PKC_DAG_Ca_GRK | 155.61 | 47.79 | 529.50 | 77.52 | (22) |
| k28ar | PKC_DAG_Ca+GRK <- PKC_DAG_Ca_GRK | 10.00 | 10.00 | 10.00 | 10.00 | (22) |
| k28bf* | PKC_DAG_Ca_GRK -> PKC_DAG_Ca+GRKp | 10.85 | 2.96 | 28.80 | 18.35 | (22) |
| k49f | DAG -> DAG_d | 0.35 | 0.35 | 0.35 | 0.35 |  |
| Vqssk50 | IP3+IP3K_a -> IP4+IP3K_a (Vmax) | 13.9 | 13.90 | 13.90 | 13.9 | (23) |
| Kqssk50 | IP3+IP3K_a -> IP4+IP3K_a (Km) | 0.0557 | 0.06 | 0.06 | 0.0557 | (23) |
| Vmaxk54 | IP4 -> IP5 | 100 | 100.00 | 100.00 | 100 | (4) |
| Kmk54 | IP4 -> IP5 | 1.4 | 1.40 | 1.40 | 1.4 |  |
| k55f | IP5 -> PIP2 | 0.008 | 0.01 | 0.01 | 0.008 |  |
| c2 | ratio of ER volume/cell: de young | 0.185 | 0.19 | 0.19 | 0.185 | (16, 17) |
| v1 | Ca channel flux constant | 1.00E+08 | 1.00E+08 | 1.00E+08 | 1.00E+08 | (16, 17) |
| v8 | leak flux constant | 0.15 | 0.15 | 0.15 | 0.15 |  |
| v4 | maximum Ca uptake rate (SERCA) | 20 | 20.00 | 20.00 | 20 |  |
| k4 | activation constant of SERCA pump | 0.65 | 0.65 | 0.65 | 0.65 |  |
| a1 | Ca leak into the cell from outside | 0.0055 | 0.01 | 0.01 | 0.0055 |  |
| Kex | Na/Ca exchange activation const | 0.25 | 0.25 | 0.25 | 0.25 |  |
| Vex | maximum Ca exchange rate | 0.023 | 0.02 | 0.02 | 0.023 |  |

# References
