# Supplementary material for: A Dual Receptor Crosstalk Model of G-Protein-Coupled Signal Transduction
Source: PLoS Comput Biol. 2008 Sep 26;4(9):e1000185. doi: 10.1371/journal.pcbi.1000185 (PMC2528964; doi:10.1371/journal.pcbi.1000185)

Table S4: Goodness of Fit Evaluation

We use the mean squared error criterion to evaluate the goodness of our model fit to the data. We have used this data in the estimation procedure and thus does not constitute a true validation. However, we show that in general our model fits the bulk of the data. Those areas of lack-of-fit are usually due to extraordinary experiment-to-experiment variation and in some cases point to unaccounted mechanisms. We elaborate on one such mechanism (multiple GRK isoforms) in the text of the article.


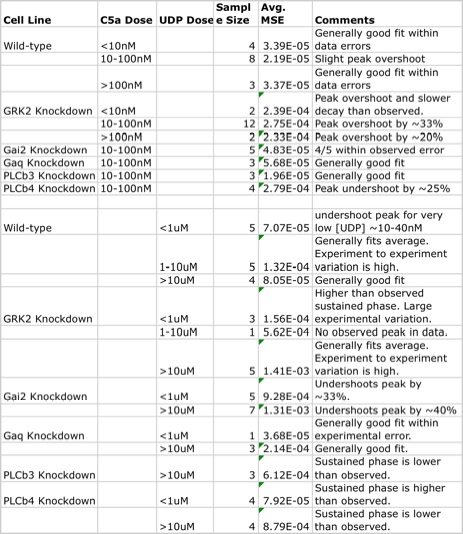

Supplement: Table S4 — Goodness of fit evaluation. We use the mean squared error criterion to evaluate the goodness of our model fit to the data. We have used this data in the estimation procedure and thus does not constitute a true validation. However, we show that in general our model fits the bulk of the data. Those areas of lack-of-fit are usually due to extraordinary experiment-to-experiment variation and in some cases point to unaccounted mechanisms. We elaborate on one such mechanism (multiple GRK isoforms) in the text of the article. (0.27 MB DOC) [file pcbi.1000185.s015.doc]
